# The Effectiveness of Public Health Interventions to Reduce the Health Impact of Climate Change: A Systematic Review of Systematic Reviews

**DOI:** 10.1371/journal.pone.0062041

**Published:** 2013-04-25

**Authors:** Maha Bouzid, Lee Hooper, Paul R. Hunter

**Affiliations:** Norwich Medical School, University of East Anglia, Norwich, Norfolk, United Kingdom; Universidade Federal do Acre (Federal University of Acre), Brazil

## Abstract

**Background:**

Climate change is likely to be one of the most important threats to public health in the coming years. Yet despite the large number of papers considering the health impact of climate change, few have considered what public health interventions may be of most value in reducing the disease burden. We aimed to evaluate the effectiveness of public health interventions to reduce the disease burden of high priority climate sensitive diseases.

**Methods and Findings:**

For each disease, we performed a systematic search with no restriction on date or language of publication on Medline, Web of Knowledge, Cochrane CENTRAL and SCOPUS up to December 2010 to identify systematic reviews of public health interventions. We retrieved some 3176 records of which 85 full papers were assessed and 33 included in the review. The included papers investigated the effect of public health interventions on various outcome measures. All interventions were GRADE assessed to determine the strength of evidence. In addition we developed a systematic review quality score. The interventions included environmental interventions to control vectors, chemoprophylaxis, immunization, household and community water treatment, greening cities and community advice. For most reviews, GRADE showed low quality of evidence because of poor study design and high heterogeneity. Also for some key areas such as floods, droughts and other weather extremes, there are no adequate systematic reviews of potential public health interventions.

**Conclusion:**

In conclusion, we found the evidence base to be mostly weak for environmental interventions that could have the most value in a warmer world. Nevertheless, such interventions should not be dismissed. Future research on public health interventions for climate change adaptation needs to be concerned about quality in study design and should address the gap for floods, droughts and other extreme weather events that pose a risk to health.

## Introduction

There is consensus that climate change is affecting human health [1]. Although the exact health impacts of climate change are still being debated these are likely to include heat stress, increased risk of vector-borne, waterborne and food-borne diseases. In addition, the increased frequency of extreme weather events such as drought, flooding or hurricanes will also have a range of public health impacts. Nevertheless, linkages between public health and climate change are complex and interact with other factors. This review is not a systematic evaluation of climate sensitive diseases; it is rather focusing on the likely adverse health impacts of climate change.

Arthropod-borne diseases are infections spread by insect (mosquitoes and sandflies) or arachnid (ticks) vectors [2]. Major shifts in the epidemiology of several vector-borne diseases and appearances on new continents have been predicted as a result of climate change [3], [4], [5], [6], [7]. Climate change is likely to increase the burden of West Nile fever, dengue, Chikungunya fever, malaria, leishmaniasis, tick-borne encephalitis, Lyme borreliosis, Crimean-Congo haemorrhagic fever, spotted fever rickettsioses, Yellow fever and Rift Valley fever [2], [7], [8], [9], [10].

Waterborne diseases are also likely to be influenced by climate change. The importance of climate as a driver of disease risk is derived from observations that waterborne disease outbreaks are often preceded by heavy rainfall [11], . This link is likely to be most obvious for inadequately treated water or small rural supplies [15], [16], [17], [18], [19]. Several authors have pointed out links between cholera and climate variables especially higher temperature and rainfall [20], [21], [22], [23], flooding [24], [25] and major climatic cycles such as El Nino [26], [27]. Risk from non-cholera *vibrios*, especially *V. vulnificus* and *V. parahaemolyticus* in marine waters, is increasing with warmer sea temperatures and higher trophic state index [28], [29], [30]. Cyanobacteria are present in drinking and recreational waters and most reported human cases were associated with observable cyanobacterial blooms [31]. The impact of droughts on health through reduced access to water in affluent countries is not clear, but effects in resource poor countries are likely to be dramatic [32], [33].

Extreme weather events pose particular challenges to populations. The frequency and intensity of extreme weather events is predicted to rise as a result of climate variability [8]. The effects of disasters such as floods, extreme droughts, storms and hurricanes on human health seem to be mostly indirect (mediated through vector and waterborne diseases), nevertheless, acute injuries, fatalities and mental health illnesses are also significant public health outcomes but their management and prevention (disaster preparedness and response planning) are beyond the scope of this study. The problems of heat stress and heat stress-related mortality are considered an area of major direct impact because of the severity of the outcome (death) and increased political sensitivity [8], [34]. The intense 2003 European heat wave caused the death of 22,000–35,000 mainly elderly persons [34], [35].

There are other diseases that are likely to be exacerbated in a warmer world including food-borne and respiratory diseases. It has been shown that reported cases of salmonellosis peak in the hot summer months and that this association was observed at temperatures greater than 7.5°C [36]. We consider the effect of climate change on food-borne diseases to be minimal providing appropriate food handling and storage procedures and improved food hygiene as previously reported by Lake and colleagues [37]. Respiratory diseases are mainly linked to air quality. Concentrations of air pollutants (mainly ozone and particulate matter) would increase with greenhouse gas emissions and higher temperatures [38]. Because the main driver of respiratory disease is air pollution itself rather than climate change, it will not be included in our high priority climate sensitive diseases. In addition, the main intervention for respiratory diseases is emission reduction, which is beyond the scope of this study.

Despite substantial peer-reviewed and gray literature investigating potential health impacts of climate change, less attention has been paid to adaptation options. While implementation of effective control interventions is the only way to reduce the disease burden of climate change, evaluation of the effectiveness of public health interventions is lacking. As the World Health Organization (WHO) stated “There is a lack of targeted, systematic reviews to identify and assess the effectiveness of interventions to control key climate-sensitive health risks, e.g. for the control of vector-borne diseases or heat health action plans” [9]. Our objective is to address this gap and systematically review existing systematic reviews on the effectiveness of public health interventions to reduce the disease burden of climate change.

## Methods

### Search methodology and inclusion criteria

Included studies were systematic reviews (defined as reviews with a specified methodology that included searches of at least 2 databases or one database plus references from at least one earlier systematic review) of any public health intervention for these climate sensitive health risks (West Nile fever, dengue, Chikungunya fever, malaria, leishmaniasis, tick-borne encephalitis, Lyme borreliosis, Crimean-Congo haemorrhagic fever, spotted fever rickettsioses, Yellow fever, Rift Valley fever, cholera, waterborne diseases, floods, droughts cyanobacteria, and heat stress) with any health related outcome measures (disease incidence/prevalence/risk, clinical manifestation, entomological indices for mosquito-borne diseases). Studies presenting primary data for interventions or assessing efficiency of therapeutic methods were excluded unless such interventions could be carried out by lay people. Where systematic reviews had been updated, only the newest version was included.

Ovid MEDLINE, ISI Web of Knowledge, Cochrane CENTRAL and SCOPUS databases were searched with no restriction on year or language of publication up to December 2010. A broad search strategy was used to improve sensitivity and to include any type of public health intervention. For each disease/issue, specific key words and/or MeSH terms were used (Table S1) and the search was combined with “systematic review” or “meta-analysis”. Reference lists from obtained articles were screened for additional relevant reviews. No protocol has been published for this systematic review.

Titles, abstracts and full texts were assessed independently for inclusion by two reviewers. Data extraction was performed in duplicate using a standardised form. Recorded information included main outcome measure, number of included studies, and effectiveness in terms of Relative Risk where provided, or Risk Ratio, odds ratio or biological indices where necessary.

### Assessment of the quality of evidence using GRADE

For each systematic review, the quality of the evidence was assessed using the GRADE method (an acronym for Grading of Recommendations: Assessment, Development and Evaluation (http://www.gradeworkinggroup.org/)). GRADE provides guidance for rating quality of evidence and grading strength of recommendations in health care and is widely used by international organisations including WHO [38], [39]. GRADE assesses the quality of a body of evidence based on 5 criteria: risk of bias, imprecision, inconsistency, indirectness of evidence and publication bias [40]. Within this review each domain was assessed based on the following scale: “no” (no risk of bias or imprecision or inconsistency or publication bias, depending on the domain) (score 0), “serious” (serious risk of bias etc.) (score −1) or “very serious” (very serious risk of bias or publication bias etc.) (score −2). The basis of assessment was

- Risk of bias – Allocation concealment, lack of blinding and incomplete accounting for fate of participants. Serious risk of bias was indicated by poor allocation concealment, blinding or follow-up, or lack of reporting of any two of these elements. Very serious risk of bias was indicated by poor, or lack of reporting of, more than one of the elements of study validity. Selective outcome reporting is also an important element of study validity, but was omitted from this assessment as it is difficult to assess and would result in almost all validity assessments suggesting serious risk of bias. In observational studies confounding and similarity of the different groups at baseline needed to be assessed.
- Imprecision – serious imprecision was assumed to occur when the review collated <300 total events (dichotomous outcomes) or a total population size <400 (continuous outcomes). Very serious imprecision occurred when there were <100 total events or a population size of <150 or where these were not reported and could not be estimated. Where the events or population were not reported, but the effect was statistically significant the evidence were assessed as at serious risk of bias. Where the number of events was not provided for dichotomous outcomes then we assumed that imprecision was serious (rather than very serious) if the review stated that there were at least 1000 participants.
- Inconsistency – Unexplained heterogeneity of results. Scored “no” where heterogeneity was not present, or where it was present but explored, and scored “serious” where heterogeneity was clearly present (stated by the reviewers or clearly observable in the forest plot) but not explored or explained, or where not reported, or where only 1 study was found (as homogeneity could not be corroborated).
- Indirectness of evidence – presence of an indirect comparison or indirect evidence (studies did not directly address the question). Where intermediate markers such as entomological parameters were measured rather than health outcomes such as actual cases of disease or disease side effects such as low serum haemoglobin and miscarriage (side effects of malaria), this was considered indirect evidence (scored as serious risk of indirectness of evidence).
- Publication bias was assessed as “undetected” (score 0) or “strongly suspected” (score −1) on the basis that it was assessed and no evidence of bias was found. Where no information was presented this was scored “strongly suspected” unless there were fewer than 10 included studies as publication bias is difficult to assess in the presence of so few studies.

The GRADE summary score was the addition of the previous scores. These scores could be upgraded by the following factors: a large effect size (RR>2 or <0.5 score +1, RR>5 or <0.2 score +2), confounders working against bias (score +1), and/or presence of a dose response (score +1).

The interpretation of these final scores were as follows: for evidence based on intervention studies a score ≥0 equates to “high quality”, −1 “moderate quality”, −2 “low quality” and ≤−3 “very low quality” and for evidence based on observational studies a score ≥+2 equates to “high quality”, +1 “moderate quality”, 0 “low quality” and ≤−1 “very low quality” evidence. For more details see Table S2.

The GRADE data were re-analysed to separate out the level of evidence of the underlying studies from the methodological quality of the reviews (as in the main GRADE assessment points could be deducted where the validity of the included studies was poor, or where the reviewer had not reported the validity of the included studies). Where points were lost for review factors (rather than due to underlying data) these were highlighted in red (see Table S2). This allowed us to also calculate the maximum possible score for the underlying evidence (assuming that the underlying studies were of high quality but this was not reported in the reviews). This “BEST” GRADE score (Table S2) was the best possible grade of the underlying studies if all un-presented study characteristics were of very high quality (extremely unlikely). The difference between the GRADE score and the best possible GRADE score was then used to determine the review quality score. A score of 0 was judged to indicate a very good systematic review, −2 ≤score<0 a good review, −3 ≤score≤−4 a poor review and score <−4 a very poor review. This approach allowed us to directly judge the quality of the systematic reviews independently from the underlying studies. In addition of highlighting high quality reviews, this approach allowed us to determine if more high quality systematic reviews are needed to clarify the evidence (where the potential level of evidence is reasonable, but the level of evidence as assessed from the present reviews is not) or if further primary studies are needed (where a really thorough review will have little or no impact on the level of evidence we have available, and the level of evidence is not great).

Data were summarised narratively with reference to tables on study characteristics and GRADE assessment and individual disease foci grouped into vector-borne diseases, waterborne diseases, cholera and heat stress.

## Results

**Figure 1:**
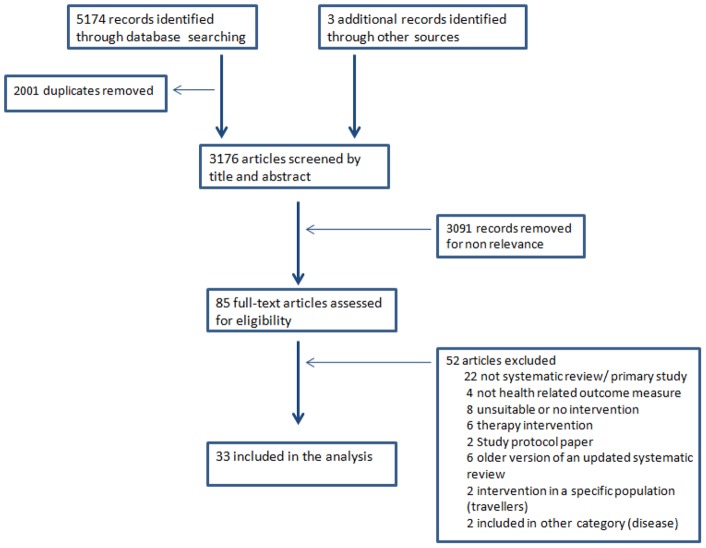
Flow diagram describing paper selection and inclusion/exclusion process according to PRISMA guidelines.

Altogether 3176 titles and abstracts were retrieved, of which 85 were assessed for inclusion in full text and 33 systematic reviews included (Figure 1). No suitable systematic reviews were found for Crimean-Congo hemorrhagic fever, Chikungunya, cyanobacteria, droughts, floods, Rift Valley fever, spotted fever rickettsioses, West Nile or Yellow fever. Appropriate systematic reviews were found for cholera, dengue, heat stress, leishmaniasis, Lyme disease, malaria, tick-borne encephalitis and waterborne diseases. Table 1 describes the characteristics of all included systematic reviews.

**Table 1:** Evaluation of the effectiveness of public health interventions to reduce the disease burden of climate sensitive diseases.

Intervention	Main outcome			References	Number of included studies	Pooled effect size: relative risk (95% CI) unless otherwise stated	GRADE summary score	Review Quality score
**Environmental interventions for control of vector-borne diseases**								
**Dengue**								
Outdoor insecticide spraying	Reduction in entomological parameters			[41]	5 (out of 19)	0.24 (0.05–1.19) (Breteau index)	−4, very low quality	−3, poor review
Biological control of the vector (e.g. larvivorous fish, predatory insect larvae, copepods)	Reduction in entomological parameters			[41]	9 (out of 10)	0.18 (0.07–0.44) (Container index)	−4, very low quality	−3, poor review
Environmental management (e.g. removal of unused water vessels and covering of water containers)	Reduction in entomological parameters			[41]	9 (out of 14)	0.71 (0.55–0.90) (Breteau index)	−5, very low quality	−3, poor review
Integrated vector management (environmental management combined with vector control)	Reduction in entomological parameters			[41]	11	0.33 (0.22–0.48) (Breteau index)	−4, very low quality	−4, poor review
Peridomestic space spraying with insecticide	Dengue incidence (new dengue cases)			[42]	1	No pooled effect size was calculated	−5, very low quality	−3, poor review
Peridomestic space spraying with insecticide	Reduction in entomological indices			[42]	15	No pooled effect size was calculated	−6, very low quality	−3, poor review
Community based dengue control programmes (education and/or insecticide spraying and/or biological control)	Entomological indices, detection of larval stages, confirmed dengue cases			[43]	11	No pooled effect size was calculated	−5, very low quality	−4, poor review
Educational or behavioural interventions	Reduction in entomological parameters			[44]	5	41.6% (range 4.0–87.6%) (percentage reduction)	−4, very low quality	−3, poor review
Biological intervention	Reduction in entomological parameters			[44]	5	96.3% (range 75.1–100%) (percentage reduction)	−2, low quality	−2, good review
Insecticide spraying	Reduction in entomological parameters			[44]	6	27.2% (range 13.9–73.8%) (percentage reduction)	−4, very low quality	−3, poor review
Community-based educational interventions	Reduction in entomological indices			[45]	22	0.25 (0.17–0.37)	−4, very low quality	−3, poor review
**Malaria**								
Environmental modification (to reduce vector habitats), long-lasting e.g. Installation/maintenance of drains		Clinical malaria variables (Combined data on malaria incidence, spleen rates & mortality)		[46]	16 (out of 27)	0.120 (0.079–0.183) for environmental modification and manipulation	−3, very low quality	−3, poor review
Environmental manipulation (temporary unfavourable vector conditions) e.g. water & vegetation management		Clinical malaria variables (Combined data on malaria incidence, spleen rates & mortality)		[46]	1 (out of 4)	No pooled effect size was calculated	−1, moderate quality	−2, good review
Modification of human habitation e.g. improving quality of houses		Clinical malaria variables (Combined data on malaria incidence, spleen rates & mortality)		[46]	8 (out of 9)	0.205 (0.128–0.326)	−3, very low quality	−3, poor review
**Leishmaniasis**								
Dog culling	Human and/or canine infection (seronconversion/clinical symptoms)			[47]	5	No pooled effect size presented	−5, very low quality	−5, very poor review
Use of insecticide	Insect density and/or leishmaniasis prevalence			[47]	4	No pooled effect size presented	−5, very low quality	−3, poor review
Combined interventions (dog culling and insecticide spraying)	Human/canine infection			[47]	4	No pooled effect size presented	−5, very low quality	−5, very poor review
Dog vaccine	Seroconversion and/or symptoms			[47]	1	No pooled effect size presented	−5, very low quality	−3, poor review
**Personal protective measures for control of vector-borne diseases (malaria)**								
Insecticide-treated mosquito nets (ITN)		Clinical malaria (*Plasmodium falciparum* parasitemia)	[48]		6	0.76 (0.62–0.94)	−6, very low quality	−6, very poor review
Insecticide-treated mosquito nets (ITN) in pregnancy		Mean haemoglobin levels (g/l)	[49]		11	0.5 (−0.95–1.95) mean difference	−1, moderate quality	−1, good review
Insecticide-treated mosquito nets (ITN) in pregnancy		Low birth weight (<2,500g)	[49]		8	0.77 (0.61–0.98)	−1, moderate quality	−1, good review
Insecticide-treated mosquito nets (ITN) in pregnancy		Miscarriages/stillbirths	[49]		4	0.67 (0.47–0.97)	−2, low quality	−2, good review
Insecticide-treated mosquito nets (ITN) in pregnancy		Placental parasitemia	[49]		5	0.77 (0.66–0.90)	−2, low quality	−2, good review
Insecticide-treated mosquito nets (ITN)		All cause child mortality (ACCM) in children <5 years	[50]		3	0.82 (0.75–0.90)	−1, moderate quality	0, very good review
Insecticide-treated mosquito nets (ITN)		Incidence of uncomplicated malaria in children <5 years	[50]		4	0.49 (0.44–0.54)	−2, low quality	−1, good review
Insecticide-treated mosquito nets (ITN)		Prevalence of malaria parasitemia (in children <5 yrs)	[50]		6	0.83− (0.64–0.88)	−2, low quality	−1, good review
Indoor residual spraying (IRS)		All cause child mortality (ACCM) in children <5 years	[50]		2	0.53 (0.40–0.71)	−3, very low quality	−1, good review
Indoor residual spraying (IRS)		All cause infant mortality	[50]		3	0.47 (0.40–0.54)	−2, low quality	−1, good review
Indoor residual spraying (IRS)		Incidence of uncomplicated malaria	[50]		3	0.25 (0.03–2.23)	−2, low quality	−1, good review
Indoor residual spraying (IRS)		Prevalence of malaria parasitemia (in children <5 years)	[50]		5	0.16 (0.03–0.94)	−1 moderate quality	−1, good review
Intermittent preventive therapy (IPT) in pregnancy		Neonatal mortality	[50]		2	0.62 (0.37–1.05)	−3, very low quality	−1, good review
Intermittent preventive therapy (IPT) in pregnancy		Perinatal mortality	[50]		1	0.83 (0.52–1.20)	−4, very low quality	−2, good review
Intermittent preventive therapy (IPT) and insecticide-treated mosquito nets (ITN) in pregnancy		Low birth weight	[50]		5	0.65 (0.55–0.77)	−2, low quality	−1, good review
Efficacy of mosquito coils to prevent malaria		Clinical malaria	[51]		0	No pooled effect size presented	No evidence	
Efficacy of mosquito coils to prevent malaria		Anti-mosquito outcome: bite inhibition	[51]		13	No pooled effect size presented	−7, very low quality	−6, very poor review
Efficacy of mosquito coils to prevent malaria		Anti-mosquito outcome: repellence	[51]		7	No pooled effect size presented	−7, very low quality	−6, very poor review
Efficacy of mosquito coils to prevent malaria		Anti-mosquito outcome: percentage of mosquito mortality	[51]		7	No pooled effect size presented	−7, very low quality	−6, very poor review
**Immunization for vector-borne diseases**								
**Malaria**								
SPf66 Vaccine vs. placebo for preventing malaria	New malaria episode (*P. falciparum*)			[52]	9	0.90 (0.84–0.96)	0, high quality	0, very good review
SPf66 Vaccine vs. placebo for preventing malaria	New malaria episode (*P. vivax*)			[52]	5	1.00 (0.93–1.08	0, high quality	0, very good review
Blood stage Vaccine vs. placebo for preventing malaria	New malaria episode			[53]	1	0.38 (0.26–0.57)	−2, low quality	0, very good review
CS-NANP Pre-erythrocytic vaccine vs. placebo for preventing malaria	New malaria episode			[54]	3	1.05 (0.82–1.35)	−1 moderate quality	0, very good review
RTS.S Pre-erythrocytic vaccine vs. placebo for preventing malaria	New malaria episode			[54]	3	0.95 (0.86–1.04)	0, high quality	0, very good review
RTS.S Pre-erythrocytic vaccine vs. placebo for preventing malaria	Clinical malaria			[54]	2	0.76 (0.66–0.88)	0, high quality	0, very good review
ME-TRAP Pre-erythrocytic vaccine vs. placebo for preventing malaria	New malaria episode			[54]	1	0.97 (0.79–1.18)	−1, moderate quality	0, very good review
**Tick-borne encephalitis**								
Vaccines for preventing tick borne encephalitis (TBE)	TBE, antibody titre and adverse effects			[55]	11	No pooled effect size was calculated because of high heterogeneity	−5, very low quality	−2, good review
**Chemoprophylaxis for vector-borne diseases**								
**Lyme disease**								
Efficacy of antibiotic prophylaxis for the prevention of Lyme disease after *Ixodes* tick bite	Development of Lyme disease symptoms and presence of anti *Borrelia* antibodies			[56]	4	0.084 (0.002–0.57) odds ratio	−3, very low quality	−1, good review
**Malaria**								
Primary chemoprophylaxis with primaquine vs. placebo	Proportion of protection (no malaria)			[58]	7	1.880 (1.327–2.179)	−4, very low quality	−4, poor review
Safety of Mefloquine vs. placebo	Tolerability (Withdrawal rate)			[59]	4	3.49 (1.42–8.56) odds ratio	−2, low quality	−1, good review
Atovaquone- proguanil (Malarone) vs. control	Parasitaemia			[60]	6	0.041 (0.020–0.082)	−1, moderate quality	−1, good review
Atovaquone- proguanil (Malarone) vs. alternate anti-malarial prophylaxis	Self- reported adverse effect			[60]	4	0.82 (0.67–1.01)	−2, low quality	−2, good review
2 dose intermittent preventive therapy (IPT) with sulfadoxine-pyrimethamine vs. placebo for malaria control during pregnancy	Placental malaria			[61]	5	0.48 (0.35–0.68)	0, high quality	0, very good review
(IPT) with sulfadoxine-pyrimethamine vs. placebo for malaria control during pregnancy	Low birth weight (<2500g)			[61]	4	0.71 (0.55–0.92)	−2, low quality	0, very good review
intermittent sulfadoxine-pyrimethamine vs. placebo for malaria control during pregnancy	Maternal anaemia			[61]	4	0.90 (0.81–0.99)	−1, moderate quality	0, very good review
IPT vs. placebo in pregnant women	Antenatal parasitaemia			[62]	2	0.53 (0.33–0.86)	−4, very low quality	0, very good review
IPT vs. placebo in pregnant women	Placental malaria			[62]	3	0.34 (0.26–0.45)	−3, very low quality	−1, good review
IPT vs. placebo in pregnant women	Severe antenatal anaemia			[62]	4	0.62 (0.50–0.78)	−2, low quality	0, very good review
IPT vs. placebo in pregnant women	Perinatal death			[62]	3	0.73 (0.53–0.99)	−2, low quality	0, very good review
IPT vs. placebo in pregnant women	Low birth weight			[62]	7	0.57 (0.46–0.72)	−2, low quality	0, very good review
Anti-malarial drugs vs. placebo for preventing malaria in children	Clinical malaria			[63]	10	0.53 (0.38–0.74)	−2, low quality	−1, good review
Anti-malarial drugs vs. placebo for preventing malaria in children	Severe anaemia			[63]	9	0.70 (0.52–0.94)	−2, low quality	−1, good review
Anti-malarial drugs vs. placebo for preventing malaria in children	Hospital admission for any cause			[63]	5	0.64 (0.49–0.82)	−2, low quality	0, very good review
**Water interventions for control of waterborne diseases**								
Relationship between diarrhoea and distance from home to water source	Self-reported diarrhoea			[33]	6	1.45 (1.04–1.68) odds ratio	−3, very low quality	−1, good review
Household chlorination of drinking water	Child diarrhoea (Self-reported)			[64]	10	0.71 (0.58–0.87)	−3, very low quality	−3, poor review
Water quality interventions	Self-reported diarrhoea			[65]	2 (out of 35)	0.39 (0.33–0.7)	−5, very low quality	−4, poor review
Impact of improvements in water quality (source and household interventions)	Self-reported			[66]	41	No pooled effect size presented	−3, very low quality	−2, good review
Water supply interventions	Self-reported diarrhoea			[67]	6	0.75 (0.62–0.91)	−5, very low quality	−5, very poor review
Water quality intervention at source	Self-reported diarrhoea			[67]	3	0.89 (0.42–1.46)	−5, very low quality	−5, very poor review
Household water treatment	Self-reported diarrhoea			[67]	12	0.65 (0.48–0.88)	−6, very low quality	−5, very poor review
Household water interventions (water treatment and storage intervention)	Onset of diarrhoea			[68]	7	No pooled effect size presented	−4, very low quality	−3, poor review
Household water interventions (water treatment & storage interventions)	Onset of cholera			[68]	3	0.35 (0.21–0.56) odds ratio	−4, very low quality	−4, poor review
Household water treatment interventions	Self-reported diarrhoea			[69]	39 intervention arms (28 studies)	0.56 (0.51–0.63)	−5, very low quality	−4, poor review
Water supply intervention at point of use	Self-reported diarrhoea			[70]	4	0.79 (0.63–0.98)	−4, very low quality	−3, poor review
Community water supply intervention at source	Self-reported diarrhoea			[70]	2	0.95 (0.90–1.00)	−4, very low quality	−3, poor review
Point of use water quality intervention	Self-reported diarrhoea			[70]	28	0.56 (0.48–0.65)	−4, very low quality	−3, poor review
Community water quality intervention at source	Self-reported diarrhoea			[70]	3	0.79 (0.62–1.02)	−4, very low quality	−3, poor review
**Immunization for waterborne diseases (cholera)**								
Vaccines for preventing cholera	Death from cholera			[71]	5	0.49 (0.25–0.93)	−2, low quality	0, very good review
Vaccines for preventing cholera	Cholera cases in Children <5 years			[71]	13	0.52 (0.42–0.65)	−1, moderate quality	−1, very good review
**Interventions for heat stress related disorders**								
Effectiveness of greening to decrease air temperature in urban areas	Day time air temperature in parks and green areas			[72]	16 studies (26 effect sizes)	−0.94°C (−0.71, −1.16) reduction in temperature	−6, very low quality	−5, very poor review
Effectiveness of greening to decrease air temperature in urban areas	Night time air temperature in parks and green areas			[72]	7 studies (12 effect sizes)	−1.15°C (−0.86, −1.45) reduction in temperature	−6, very low quality	−5, very poor review
Heat health warning systems	Public awareness of extreme heat episode, changes in practices and reduction of mortality and morbidity			[73]	14 (observational)	No meta-analysis was performed	−7 for heat episodes and practice, very low quality, −6 for mortality, very low quality	−6, very poor review (for heat episodes and practice) −6, very poor review (for mortality)

### Vector-borne diseases

24 systematic reviews concerned prevention of vector-borne diseases (15 about malaria, 5 dengue fever, 2 leishmaniasis and one each Lyme disease and tick-borne encephalitis). Chemoprohylaxis (malaria 7, Lyme disease 1) and immunization (malaria 3, leishmaniasis 1, tick-borne encephalitis 1) and vector control measures (10, through reducing density of insect vectors, providing barriers between susceptible humans and vectors) were common topics for review. One review focused on culling of host species).

#### Environmental interventions for control of vector-borne diseases

Environmental interventions aiming to reduce mosquito populations are of particular interest as their findings are relevant to several vector-borne diseases. The reviews under this heading relate to dengue fever, malaria and leishmaniasis.

For the five dengue reviews, the efficacy of interventions was calculated as the ratio of entomological indices in the intervention and control groups. There was considerable overlap in the included studies between the five dengue reviews. The most comprehensive review was by Erlanger et al. [41] including 56 publications covering 61 interventions. The authors identified 19 studies on chemical control of vectors. Pooled effects were only calculated for five studies that used outdoor adulticiding and reported their results as Breteau Index (BI) (number of containers with *Aedes* spp. larvae per 100 houses). Ten studies assessed biological control, of which 9 were included in a pooled analysis. The one study not included showed increased dengue risk in the intervention arm. GRADE suggested low to very low quality evidence for both chemical and biological interventions, and scores were low partly because study validity was not reported and heterogeneity was not explored or explained in the reviews (such reporting may have raised the score, or may not if the validity of included studies was low).

The review included 14 environmental management interventions such as removing unused water containers and covering used ones and the authors conducted three pooled analyses according to the outcome measure with: 9 interventions reporting BI, 10 interventions reporting container index (CI) and 10 studies reporting house index (HI). Finally, this review included 18 integrated interventions, 13 combined environmental with chemical and 5 environmental with biological. The authors reported pooled effect analyses of combined interventions for all three entomological indices. GRADE suggested that the quality of evidence for integrated vector management was very low. Scores for each of the five GRADE criteria are detailed in Table S2. Erlanger et al. [41] concluded that dengue vector control is effective in reducing vector populations. However, as indicated by GRADE scores, such a conclusion is not supported by the quality of evidence. The main problem is that no consideration was given to study quality or design and the impact of this on pooled effect size. The authors did assess publication bias, and found evidence of it for some analyses, but no attempt was made to adjust the pooled effects. The authors investigated the sources of heterogeneity but only through subgroup analysis for intervention type. Finally, because pooled analyses excluded some studies, the authors did not attempt to investigate if this would bias their conclusions. As entomological parameters (which may or may not relate strongly to health) were assessed, the outcomes were considered to be indirect, which also reduced the validity of the evidence.

Esu et al. [42] focussed on effectiveness of peridomestic insecticide spraying. They included 15 studies some of which were included in earlier reviews. The authors found that many studies were of poor quality and few took account of possible confounders. This is in accordance with GRADE which suggested very low quality evidence. No meta-analysis was reported, presumably because of the poor comparability of studies. In a narrative analysis the authors concluded that the evidence for any value of peridomestic space spraying was weak as only some studies showed an effect, which was not sustained, with mosquito populations returning to the same level or higher within few weeks.

Heintze et al. [43] focussed on community-based control programmes and included 11 studies. The authors found that most studies were of low quality and concluded that the evidence of the effectiveness of community-based dengue control programmes is weak, which was supported by GRADE summary score. Ballenger-Browning and Elder focussed on “multi-modal” mosquito reduction interventions (integrating more than one type of intervention) [44]. They identified 21 studies of which three were cluster randomised controlled trials (cluster RCTs), two were RCTs, three were interrupted time series and 13 were non-randomised controlled trials. The effect of five studies of behavioural interventions, 5 of biological interventions and 6 of chemical interventions was investigated. Many studies were also included in Erlanger et al. [41]. However, the authors did not present a meta-analysis and concluded that little evidence exists to support the efficacy of mosquito abatement programs [44], in accordance with GRADE score.

Al-Muhandis and Hunter focused on the role of community education interventions [45]. They included 22 studies and reported a pooled relative effectiveness of 0.25 (95%CI 0.17–0.37). The authors reported substantial heterogeneity but no significant publication bias, and investigated causes of heterogeneity using multi-level modelling. They found that 61% of heterogeneity could be explained by two variables (whether contemporary or historic controls were used and the time from intervention to assessment). Studies using historic controls substantially over-estimated intervention effectiveness compared to studies using concurrent control groups. When restricted to those studies with contemporary controls, educational interventions still appeared to be effective, but effectiveness declined after 18 months. There was no additional value of combining educational with chemical or biological interventions. GRADE suggested very low quality evidence.

Most reviews of dengue fever were considered of poor quality, this suggests that before further trials are commissioned in this area, high quality systematic reviews of the evidence are required.

For malaria Keiser et al. reviewed environmental measures aimed at reducing disease transmission [46]. They identified 40 studies, of these, 27 assessed effects of environmental modification such as drainage, filling-in ponds and pools and river boundary modification. Four studies assessed effects of environmental manipulation (e.g. intermittent irrigation) and 9 modification or manipulation of human habitation (e.g. mosquito proofing homes). The reviewers reported significant heterogeneity and evidence of publication bias but presented the results of meta-analyses and concluded “malaria control programmes that emphasise environmental management are highly effective in reducing morbidity and mortality”. The authors took little account of study quality, stating that it was impossible to scrutinise the methodological quality as most studies were implemented 50–100 years ago. GRADE assessment suggested the quality of evidence was moderate for environmental manipulation and very low for the other intervention types, methodological issues included use of historical controls (before-after studies) in many studies and not accounting for possible confounding factors (additional treatments or personal protection interventions). The authors' conclusion that environmental management is highly effective in reducing morbidity and mortality is not substantiated by the evidence. A further high quality review of the evidence would be helpful before commissioning further primary research in this area.

One systematic review addressed the control of visceral leishmaniasis [47] and identified 14 intervention studies, 5 of which concerned culling seropositive dogs, 4 insecticide use, 4 combined culling and insecticides and one vaccinating dogs. Outcome measures varied, including both canine and human infection rates, making meta-analysis difficult. The authors adequately considered the studies strengths and limitations, concluding there was no strong evidence for a significant impact of any of the interventions reviewed, concurring with the GRADE assessment of very low quality evidence. Additionally, good quality reviews are needed.

#### Personal protective measures for control of vector-borne diseases

Personal protection measures include use of bed nets, mosquito coils, immunization and pharmacological prophylaxis. Three reviews considered the effectiveness of insecticide-impregnated bed nets to control malaria. Choi et al. identified 22 field trials of which 6 were included in a meta-analysis comparing malarial infection in people using permethrin-impregnated bed nets versus untreated bed nets, and 6 versus no bed nets [48]. Studies were omitted from meta-analysis for poor study design, possible confounding or different outcome measures. Permethrin-impregnated bed nets compared to untreated bed nets reduced the risk of parasitaemia (RR 0.76, 95%CI 0.62 to 0.94), while permethrin-impregnated nets compared to no bed nets reduced the risk further (RR 0.49, 95%CI 0.42 to 0.60). The level of evidence as assessed by GRADE was of very low quality as validity of included studies was assessed but not reported, heterogeneity apparent in forest plots was not reported or discussed and publication bias was not reported.

More recent systematic reviews of bed net use have focussed on pregnant women and children. Gamble et al. reported on low birth weight, miscarriages and stillbirths and placental parasitaemia and found consistent protective effects of RCTs of bed nets [49]. As the included studies were assessed for validity, showed little heterogeneity of effect and included large numbers of participants, GRADE suggested moderate to very low quality evidence. Eisele et al. included cluster RCTs assessing effects of insecticide-treated mosquito nets on childhood mortality (3 RCTs), incidence of uncomplicated malaria (4 RCTs) and prevalence of malarial parasitaemia (6 RCTs) [50]. Use of insecticide-treated bed nets was associated with health gains in all three outcomes but as the validity of the included RCTs was not assessed, the GRADE level of evidence was of very low quality. Eisele at al. meta-analysed 7 before-after studies for indoor residual spraying on all-cause childhood mortality (3 studies), incidence of uncomplicated malaria (3 studies) and prevalence of malarial parasitaemia (5 studies) [50]. As reported by the reviewers, the evidence had serious limitations and was of very low to moderate quality. Their review was considered of good quality, therefore, the level of evidence can only be improved by conducting new trials.

Lawrence and Croft included 15 controlled trials of mosquito coils, using a variety of outcomes [51]. As they found no clinical malaria outcomes, they concluded that there was no evidence that burning mosquito coils prevents malaria acquisition, but they reported that such coils inhibit nuisance biting. The quality of evidence was very low.

#### Immunization for vector-borne diseases

Three Cochrane reviews by Graves and Gelband assessed vaccination for malaria. One review including 10 RCTs and quasi-RCTs of SPf66 vaccine (against the blood (asexual) stage of the malaria parasite) concluded that SPf66 was not protective against new malarial episodes of *P. falciparum* in Africa (RR 0.98, 95% CI 0.90 to 1.07) but modestly protective in South America (RR 0.72, 95% CI 0.63 to 0.82) [52]. GRADE showed high quality evidence. One review included five trials of MSP/RESA vaccine (also against parasitic blood stages), which showed promise (RR of new malarial episodes 0.38, 95% CI 0.26 to 0.57) but the results were difficult to interpret due to small numbers of new malarial episodes and lack of statistically significant effects [53]. One review of nine trials of vaccines targeted at the sporozoite or liver stages (CS-NANP, CS102, ME-TRAP and RTS, S) found that only (RTS, S) reduced clinical episodes of malaria by 26% (95% CI 13% to 37%) in semi-immune children for up to 18 months [54]. The quality of evidence was moderate to high. The protective effect was reduced in adults, and RTS, S was less efficient in preventing new malaria infections in children and adults, 6% and 4%, respectively with moderate quality evidence. The high quality reviews in this area suggest that where evidence is unclear or of low quality further trials are required to address relevant questions.

Demicheli et al. reviewed tick-borne encephalitis vaccine [55] in a Cochrane collaboration review including 11 trials. They concluded that the vaccine was highly immunogenic but that the relationship between seroconversion and clinical protection is not clear. The quality of evidence was very low as the review included trials with unclear allocation concealment and without blinding, found high levels of heterogeneity and no study reported a tick-borne encephalitis case. Further trials are needed to address this issue.

#### Chemoprophylaxis for vector-borne diseases

Eight reviews addressed the value of pharmacological interventions for the prevention of vector-borne diseases, one for Lyme disease and the rest for malaria. The Lyme disease review included four placebo-controlled RCTs of post-exposure prophylaxis using penicillin, amoxicillin, tetracycline or doxycycline [56]. The authors found a significant reduction in the odds of developing Lyme disease (OR 0.084, 95% CI 0.002 to 0.57), but the evidence was of very low quality as there were only 13 cases of Lyme disease. Therefore, further trials are needed.

Amongst seven Malaria reviews, one was superseded by others and will not be discussed [57]. Three reviews assessed the effectiveness of specific prophylactic agents for malaria: primaquine [58], mefloquine [59] and atovaquone-proguanil [60], all finding that the prophylactic agent was highly effective at reducing malaria risk. The quality of evidence was very low for primaquine as validity of the included controlled studies was not assessed and heterogeneity apparent in the forest plot was not mentioned or explored [58]. The efficacy of atovaquone-proguanil in reducing parasitaemia (RR 0.04, 95%CI 0.02–0.08) [60] was supported by moderate quality evidence. No pooled effect size was calculated for mefloquine because of the different study designs in the few field trials that reported on efficacy [59]. This was supported by low quality GRADE score.

Two reviews concerned the prevention of malaria in pregnant women. One focussed on the impact of sulfadoxine-pyrimethamine resistance in intermittent preventive therapy (IPT) [61]. The authors concluded that 2- dose IPT during pregnancy benefits HIV-negative women in preventing placental malaria (RR 0.48, 95% CI 0.35 to 0.68), low birth weight (RR 0.71, 95% CI 0.55 to 0.92), and maternal anaemia (RR 0.90, 95% CI 0.81 to 0.99). The GRADE score was high for placental malaria prevention and moderate for the other outcomes. Garner and Gülmenezoglu assessed effects of any preventive chemoprophylaxis or IPT drugs vs. no drugs in pregnant women and identified 16 studies [62] concluding that antimalarial drugs reduced severe anaemia (RR 0.62, 95%CI 0.50 to 0.78), perinatal deaths (RR 0.73; 95%CI 0.53 to 0.99) and low birth weight (RR 0.57; 95%CI 0.46–0.72). GRADE score showed low quality of evidence.

A Cochrane review by Meremikwu et al included 11 trials of prophylaxis with either chloroquine or pyrimethamine-dapsone and 10 trials of IPT in children [63]. Seven trials used sulfadoxine-pyrimethamine, one sulfadoxine-pyrimethamine and one amodiaquone. IPT or prophylaxis was associated with fewer episodes of clinical malaria and less severe anaemia, however, the quality of evidence was very low and low, respectively.

Further trials are needed to improve the level of evidence of malaria chemoprophylaxis.

### Waterborne Diseases

#### Water interventions

Eight systematic reviews assessed the impact of water interventions on self-reported diarrhoea [33], [64], [65], [66], [67], [68], [69], [70]. There was substantial overlap in primary studies included with later reviews including more recent studies. Most reviews focussed on household water interventions in developing countries, including chlorination, solar disinfection and filtration. Reviews focussing on household chlorination [64], assessing effects on diarrhoea and clinical cholera [68] and observational studies linking self-reported diarrhoeal disease to distance from home to water source [33], suggested pooled reductions in diarrhoeal disease of 30 to 50% or pooled Relative Risk or Odds Ratios of 0.5 to 0.7 for household interventions, but the GRADE quality of evidence was very low. The main issues are use of self-reported diarrhoea in unblinded intervention trials, significant heterogeneity in effect sizes and evidence of publication bias. Whilst most authors commented on heterogeneity, only two sought to investigate heterogeneity sources [69], [70]. Waddington used subgroup analyses, whereas Hunter used meta-regression. Hunter found that whether or not the study was blinded, intervention type, duration of follow-up and whether or not the intervention was conducted in an emergency setting explained 90% of the heterogeneity [69]. He concluded that most household water treatment interventions have little or no public health value and that their apparent effectiveness is due to poor study design (lack of blinding and very short follow-up periods). He suggested that ceramic filters were more effective than other technologies, but quality of evidence was very low.

Two reviews reported on community water supply interventions and found that their impact was weak [66], [70]. No conclusions about the value of community water interventions can be made in developing country settings. A review investigating the relationship between distance to fetch water and self-reported diarrhoea found an association between increased distance and increased risk [33], but this is not definitive due to poor quality studies as reflected by very low quality of evidence. In this area further high quality reviews are needed before further trials are commissioned.

#### Immunization for waterborne diseases

One review considered the value of injected whole cell or subunit vaccines in cholera prevention [71]. The authors found 16 trials involving over 1 million participants, reporting reduced risk of death from cholera (RR 0.49, 95%CI 0.25 to 0.93) and reduced risk of contracting cholera at 12 months for children <5 years old (RR 0.52, 95%CI 0.42 to 0.65). The evidence was of moderate quality. The authors concluded that injected cholera vaccines are safe and more effective than generally realised but that injected vaccines have been superseded by oral ones.

### Heat stress

Two reviews related to heat stress were found. A review of the impact of green spaces within cities found _∼_1°C lower temperatures in city parks than in built up city areas [72]. The GRADE score showed very low quality of evidence. Whether such green spaces had any impact on reducing morbidity and mortality during heat waves was not addressed. The second review included 14 observational studies investigating whether heat health warning systems increased awareness and reduced mortality and morbidity [73]. The authors presented a narrative synthesis and did not judge study validity. They reported high levels of awareness about the public health campaigns and heat wave events amongst the general public, but evidence of behavioural change as a result of this awareness was less forthcoming. The associated quality of evidence was considered to be of very low quality. A key problem was that some studies recruited people in the street and so did not include the group particularly vulnerable to heat stress. Most of the studies made comparisons between different heat wave periods in the same city before and after the heat health warning system was in place. Generally they found reduced mortality in the second period, which was attributed to the public health response. However, as pointed out by the authors, few studies considered other possible factors that may have had an impact. For example in Chicago, there had been a failure in the electricity supply during the first heat wave which would have exacerbated the adverse impact. In addition, harvesting or mortality displacement is an important factor that is likely to be a major contributor to the reduced mortality associated with the second heat wave, but this has not been considered. Bassil and Cole concluded that limited evidence suggests a positive impact of public health interventions for heat waves but the most vulnerable groups are not being adequately reached [73]. Further primary research is needed to address effective interventions for heat stress.

## Discussion

In this study, we aimed to identify high priority climate sensitive health threats that are likely to be exacerbated in a warmer world. We are aware that health impacts of climate change are highly dependent on location, economical status, infrastructure, health services (to name just a few). The main climate sensitive diseases are West Nile fever, dengue fever, Chikungunya fever, malaria, leishmaniasis, tick-borne encephalitis, Lyme borreliosis, Crimean-Congo haemorrhagic fever, spotted fever rickettsioses, Yellow fever, Rift Valley fever, cholera, waterborne diseases, floods, droughts, cyanobacteria, and heat stress. Subsequently, we looked for any intervention that is directed against these diseases with any health related outcome measure.

### Interventions for vector-borne diseases

Immunization and chemoprophylaxis have the strongest evidence for prevention of vector-borne diseases, however, these are limited to single infections. Although insecticide-impregnated bed nets are effective, their action is limited to night flying mosquitoes and can be less efficient for dengue fever and Chikungunya vectors. Immunization programmes are a long way away for these diseases, especially dengue fever, because of concerns that immunization may increase the risk of dengue haemorrhagic fever [74]. Consequently, control of these diseases will rest largely on vector control strategies.

Most systematic reviews for control of vector-borne diseases focused on the effect of environmental interventions on entomological indicators. Undeniably, vector presence and survival is key in disease transmission, however, reduction of mosquito populations does not necessarily translate in decreased disease risk. In fact, several such papers have been downgraded using GRADE for indirectness as they were not assessing health outcomes. Our recommendation is that future studies of the effect of environmental interventions for the control of vector-borne diseases should be of acceptable duration and should report primarily on disease related outcome measures.

Assessment of the strength of evidence for the effectiveness of vector control strategies depends on which review one reads. The reviews by Keiser et al. [46] on malaria and Erlanger et al. [41] on dengue control strongly support the effectiveness of such interventions, in sharp contrast with other reviews. Heintze et al. [43] concluded “Evidence that community-based dengue control programmes alone and in combination with other control activities can enhance the effectiveness of dengue control programmes is weak”. Ballenger-Browning and Elder [44] said “Little evidence exists to support the efficacy of mosquito abatement programs owing to poor study designs and lack of congruent entomologic indices” and Esu et al. [42] stated “Based on a comprehensive search of available peer reviewed literature, the effectiveness of peridomestic space spraying in reducing dengue transmission has not been conclusively demonstrated”.

Why do conclusions on the strength of evidence conflict so strongly? Principally this has to do with study quality. Although Keiser et al. [46] and Erlanger et al. [41] mentioned study quality, they did not account for this when drawing their conclusions. Indeed, Keiser et al. stated that assessments of study quality were not possible as studies were conducted over 50 years ago [46]. We consider that problems with study design and lack of control for confounding were obvious in these studies making them of low quality and reducing the value of the conclusions drawn from the meta-analyses.

Al-Muhandis and Hunter [45] used multi-level meta-regression to assess the impact of effect modifiers. It should be stated that such studies do not have the evidential standard of other meta-analyses but rather of observational studies. However, 60% of the heterogeneity in outcome measures could be explained by whether or not studies used historic or contemporary controls and time from intervention to assessment. In particular studies that used historic controls (before/after studies) substantially over-estimated effectiveness compared to studies using contemporary controls, and the over estimation increased with time from intervention to assessment. When analyses were restricted to studies with contemporary controls, the impact was more modest. The use of historic controls is considered poor practice as most historical control groups are compromised [75], [76]. Given that many studies in Keiser et al. and Erlanger et al. were before/after studies, this would also explain the substantial over-estimation of the value of such interventions [41], [46].

### Interventions for waterborne diseases

The impact of climate change on waterborne diseases in wealthy countries, relying on well-maintained water treatment plants, is likely to be negligible. The disease burden will fall largely on those reliant on small systems with inadequate treatment and intermittent supply, for which household water treatment could be an important public health intervention for adaptation to climate change. However, evidence in favour of such interventions is weak and divergent between studies. Hunter suggests that household water filtration with ceramic filters has a sustainable public health value [69]. However, this observation springs from meta-regression and so carries the weight of an observational study. For firm conclusions to be drawn there is a need for properly conducted double blinded trials on water filters. Evidence of reduction of diarrhoeal diseases after household water interventions was mainly derived from non-blinded interventions. Schmidt and Cairncross reported that when analyses were restricted to blinded studies no effect was demonstrable [77]. They suggested that the apparent beneficial effect was due to “courtesy bias” where people who received the intervention were less likely to report illness. The value (or otherwise) of household water treatment is still a topic of considerable debate. The evidence does support household water treatment for the prevention of cholera, though this was based on only three studies [68]. Explanations for the lack of effectiveness of some interventions in developing countries have been discussed previously [78]. Part of the problem is that chlorination alone is not effective against *Cryptosporidium* and has reduced impact against *Giardia* (two particularly important waterborne pathogens). A study of small systems in France showed that chlorination alone did not remove risk of illness associated with contaminated supplies, even when indicator organisms were inactivated [79]. However, the main problem appears to be inconsistent use. Arnold and Colford showed that the use of SODIS (a solar disinfection system) declines quickly after a campaign [64]. Furthermore, Hunter et al. showed that even occasional days when people revert to drinking untreated water are sufficient to undermine most of the public health gains [80].

### Interventions for Heat stress

We found one systematic review concerned with the effect of green spaces on air temperature and reported a slight cooling effect. However, the public health implication of such intervention is not clear. Nevertheless, this kind of intervention may have several co-benefits. Greening a city for example can improve air quality, promote physical activity, protect against sunlight exposure, enhance storm water management and increase property values, which are not all health related and certainly could not be accounted for using GRADE method.

The main public health intervention for Heat stress is heat health action plans. Bassil and Cole suggested that particularly vulnerable sub-groups were inadequately reached by heat health action plans [73]. A meta-analysis of observational studies seeking to identify factors associated with increased disease risk from heat waves allowed to identify this group: elderly people who are unable to care for themselves and are house-bound and/or with pre-existing medical conditions such as psychiatric, cardiovascular and pulmonary illness [81]. Abrahamson et al. interviewed 73 elderly men and women (>72 years old) living at home in London and Norwich about their perceptions of the risks from excessive heat and found that the majority were aware of the dangers of a heat wave in others, but did not think that they themselves are at risk (despite some of them suffering from relevant chronic diseases) [82]. Bassil and Cole concluded there is a high level of heat risk awareness, but this does not necessarily translate into behavioural change because of faulty self-perception of vulnerability [73].

Despite no strong evidence supporting public information ahead of heat waves reducing mortality, the costs of such public health interventions are almost negligible and so we would argue that public alerts still form part of the response to heat wave. However, the issue remains as to how the most vulnerable can be reached. Clearly public alert announcements cannot be the sole public health strategy and research needs to be targeted at protecting the vulnerable group.

For each intervention type, we assessed the quality of evidence using the GRADE system, based on five criteria (risk of bias, imprecision, inconsistency, indirectness of evidence and publication bias) each individually assessed, to come up with an overall GRADE score. This quality assessment should allow an objective judgement of the validity of the conclusions reached by the authors of the systematic reviews. While GRADE assessment is widely used by international organisations and in peer-reviewed literature, it only accepts RCTs as high quality evidence. As Guyatt et al. state “Those applying GRADE to questions about diagnostic tests, to public health, or to health systems questions will face some special challenges” [39]. Nevertheless, despite this concern, Guyatt et al. state “The GRADE system can, however, also be applied to public health …”. Since the majority of the public health interventions assessed in this study were of environmental nature and cannot be randomised, GRADE assigned them as low quality evidence. We tried to counterbalance this limitation by adopting a slightly different scaling system for intervention and observational studies. In any event, the use of the GRADE approach is still a key recommendation of WHO policy formulation. This is particularly relevant considering that one key aspect of climate policy is the concept of co-benefits, when an intervention yields multiple benefits which may or may not be included in the main outcome measures. GRADE does not allow us to account for such full benefits evaluation. Nevertheless it should still be used as a part of multi-level grading system for quality of evidence and strength of recommendations. Nevertheless, the limitation of the GRADE system should be considered especially when evaluating different types of public health interventions as we did here.

In addition, we found that the systematic reviews we included differed in quality from one to another. Consequently a low GRADE score could indicate a good quality review of poor quality primary studies or a poor quality review of studies of uncertain quality. In order to assess this we created a scoring system to grade the quality of a review independently of the quality of the underlying studies. This we called the review quality score, which was the difference in GRADE score and the best possible GRADE score assuming that all un-presented study characteristics were indicative of high quality evidence. It should be stated very clearly that evidence from a systematic review with a poor calculated GRADE score and excellent quality Best GRADE score is still poor quality evidence and should not be used to influence practice without further consideration. What this approach does do is help identify those areas with low quality of evidence within published systematic reviews that may benefit from a more thorough analysis.

In this study, we attempted to systematically review and assess the quality of evidence of public health interventions to reduce the burden of climate sensitive diseases. One of the main findings was that for several diseases, most or all of the primary studies were undertaken in the developing word. This was particularly the case for vector-borne diseases where currently the major burden falls in tropical and sub-tropical countries. Undeniably, access to resources is an important determinant of climate change adaptation and so one should be very careful when extrapolating public health research from developing to developed countries and vice-versa. However, this is not a reason for ignoring the lessons for the developed world from research conducted in developing countries, especially when research in the developed world is weak.

### Synthesis

As discussed above, the World Health Organization has called for systematic reviews on the value of public health measures aimed at reducing the impact and public health effects of climate change [9]. In this systematic review of systematic reviews, we have shown that several systematic reviews already exist for some diseases likely to be more of a threat in a warmer world, while for others there are none. The areas with the most pressing need for evidence are in the management of drought, floods, air pollution and food safety. The likely reason for this is the scarcity of primary studies as indeed was identified in our first screen (table S1). This result is not perhaps surprising. By their nature, drought and flood events are difficult to predict and when they occur most agencies are concerned primarily with responding rather than conducting research. Nevertheless, if we are to improve our ability to manage them, then we need to consider how best to improve the quality of the evidence base. This may require the development of groups of researchers able to get funding and ethical clearance very rapidly following extreme events.

A particular concern is that systematic reviews including overlapping studies have come to very different conclusions. Examples include the systematic reviews on water quality and diarrhoeal disease and also the reviews on the impact of dengue control on entomological indices. Despite the wide availability of guidance documentation on how to conduct systematic reviews, it is of great concern that researchers can review essentially the same primary research literature and come to starkly different conclusions. It would appear that such different conclusions are drawn partly because of the importance reviewers gave to the issue of study quality or heterogeneity when drawing their conclusions. It is also possible that some research synthesisers may not be as dispassionate about the conclusions of their review as they would have the reader believe, falling into the trap of confirmational bias. We strongly recommend that systematic reviews in this area should report fully on the validity of the included studies (whether interventional or observational), assess and examine heterogeneity and publication bias, report on health outcomes and state numbers of health events as well as population size. This will allow readers to truly understand the strength of evidence presented. Additionally systematic reviews and meta-analyses should include explicit GRADE scoring by an independent scorer to reflect the quality of evidence, especially when assessing effectiveness of public health interventions. This would be valuable for informing stakeholders and policy makers and should assist future policy options for climate change adaptation.

## Supporting Information

Table S1 **Summary of search and Index terms for each public health disease/area, the number of identified papers and papers included in analysis.**
(DOCX)

Table S2 **GRADE assessment of level of evidence for public health interventions to reduce the health impact of climate change.**
(DOC)

## References

[1] UNFCC (1992) United nation framework convention on climate change. http://unfccc.int/resource/docs/convkp/conveng.pdf. Accessed 2013 March 24.

[2] Hunter PR (2003) Climate change and waterborne and vector-borne disease. J Appl Microbiol 94 Suppl: 37S–46S 10.1046/j.1365-2672.94.s1.5.x 12675935

[3] Elliott RM (2009) Bunyaviruses and climate change. Clin Microbiol Infect 15: 510–517. 19604275 10.1111/j.1469-0691.2009.02849.x

[4] Hunter PR (2011) Vector borne disease and climate change. In: Nriagu JNJ, editor. Encyclopedia of Environmental Health: Elsevier.

[5] Reiter P (2010) West Nile virus in Europe: understanding the present to gauge the future. Euro Surveill 15: 19508. 20403311

[6] Rogers DJ, Randolph SE (2006) Climate change and vector-borne diseases. Adv Parasitol 62: 345–381. 16647975 10.1016/S0065-308X(05)62010-6

[7] Semenza JC, Menne B (2009) Climate change and infectious diseases in Europe. Lancet Infect Dis 9: 365–375. 19467476 10.1016/S1473-3099(09)70104-5

[8] McMichael AJ, Woodruff RE, Hales S (2006) Climate change and human health: present and future risks. Lancet 367: 859–869. 16530580 10.1016/S0140-6736(06)68079-3

[9] World Health Organization (2009) Protecting health from climate change: Global research priorities. Available: http://www.who.int/globalchange/publications/9789241598187/en/index.html. Accessed 2013 Mar 24.

[10] DH (2008) Health Effects of Climate Change in the UK 2008: An update of the Department of Health Report 2001/2002. Available: http://www.dh.gov.uk/prod_consum_dh/groups/dh_digitalassets/dh/en/documents/digitalasset/dh_082836.pdf. Accessed 2013 Mar 24.

[11] Curriero FC, Patz JA, Rose JB, Lele S (2001) The association between extreme precipitation and waterborne disease outbreaks in the United States, 1948–1994. Am J Public Health 91: 1194–1199. 11499103 10.2105/ajph.91.8.1194 PMC1446745

[12] Hrudey SE, Payment P, Huck PM, Gillham RW, Hrudey EJ (2003) A fatal waterborne disease epidemic in Walkerton, Ontario: comparison with other waterborne outbreaks in the developed world. Water Sci Technol 47: 7–14. 12638998

[13] Nichols G, Lane C, Asgari N, Verlander NQ, Charlett A (2009) Rainfall and outbreaks of drinking water related disease and in England and Wales. J Water Health 7: 1–8. 18957770 10.2166/wh.2009.143

[14] Risebro HL, Doria MF, Andersson Y, Medema G, Osborn K, et al (2007) Fault tree analysis of the causes of waterborne outbreaks. J Water Health 5 Suppl 11–18. 10.2166/wh.2007.136 17890833

[15] Confalonieri U, Menne B, Akhtar R, Ebi KL, Hauengue M, et al.. (2007) Human health. Climate Change 2007: Impacts, Adaptation and Vulnerability. 391–431. p.

[16] Kay D, Watkins J, Francis CA, Wyn-Jones AP, Stapleton CM, et al (2007) The microbiological quality of seven large commercial private water supplies in the United Kingdom. J Water Health 5: 523–538. 17878565 10.2166/wh.2007.042

[17] Lake IR, Bentham G, Kovats RS, Nichols GL (2005) Effects of weather and river flow on cryptosporidiosis. J Water Health 3: 469–474. 16459850 10.2166/wh.2005.048

[18] Naumova EN, Christodouleas J, Hunter PR, Syed Q (2005) Effect of precipitation on seasonal variability in cryptosporidiosis recorded by the North West England surveillance system in 1990–1999. J Water Health 3: 185–196. 16075943

[19] Richardson HY, Nichols G, Lane C, Lake IR, Hunter PR (2009) Microbiological surveillance of private water supplies in England: the impact of environmental and climate factors on water quality. Water Res 43: 2159–2168. 19303126 10.1016/j.watres.2009.02.035

[20] Hashizume M, Faruque AS, Wagatsuma Y, Hayashi T, Armstrong B (2010) Cholera in Bangladesh: climatic components of seasonal variation. Epidemiology 21: 706–710. 20562706 10.1097/EDE.0b013e3181e5b053

[21] Islam MS, Sharker MA, Rheman S, Hossain S, Mahmud ZH, et al (2009) Effects of local climate variability on transmission dynamics of cholera in Matlab, Bangladesh. Trans R Soc Trop Med Hyg 103: 1165–1170. 19477477 10.1016/j.trstmh.2009.04.016

[22] Luque Fernandez MA, Bauernfeind A, Jimenez JD, Gil CL, El Omeiri N, et al (2009) Influence of temperature and rainfall on the evolution of cholera epidemics in Lusaka, Zambia, 2003–2006: analysis of a time series. Trans R Soc Trop Med Hyg 103: 137–143. 18783808 10.1016/j.trstmh.2008.07.017

[23] Paz S (2009) Impact of temperature variability on cholera incidence in southeastern Africa, 1971–2006. Ecohealth 6: 340–345. 20039097 10.1007/s10393-009-0264-7

[24] Hashizume M, Wagatsuma Y, Faruque AS, Hayashi T, Hunter PR, et al (2008) Factors determining vulnerability to diarrhoea during and after severe floods in Bangladesh. J Water Health 6: 323–332. 19108552 10.2166/wh.2008.062

[25] Siva N (2010) Pakistan sees first suspected cases of cholera. BMJ 341: c4525. 20719853 10.1136/bmj.c4525

[26] Kovats RS, Bouma MJ, Hajat S, Worrall E, Haines A (2003) El Nino and health. Lancet 362: 1481–1489. 14602445 10.1016/S0140-6736(03)14695-8

[27] Ohtomo K, Kobayashi N, Sumi A, Ohtomo N (2010) Relationship of cholera incidence to El Nino and solar activity elucidated by time-series analysis. Epidemiol Infect 138: 99–107. 19538819 10.1017/S0950268809990203

[28] Baker-Austin C, Stockley L, Rangdale R, Martinez-Urtaza J (2010) Environmental occurrence and clinical impact of Vibrio vulnificus and Vibrio parahaemolyticus: a European perspective. Environmental Microbiology Reports 2: 7–18. 23765993 10.1111/j.1758-2229.2009.00096.x

[29] Paerl H, Huisman J (2009) Climate change: a catalyst for global expansion of harmful cyanobacterial blooms. Environmental Microbiology Reports 1: 27–37. 23765717 10.1111/j.1758-2229.2008.00004.x

[30] Savage C, Leavitt P, Elmgren R (2010) Effects of land use, urbanization, and climate variability on coastal eutrophication in the Baltic Sea. Limnol Oceanogr 55: 1033–1046.

[31] Hunter PR (1998) Cyanobacterial toxins and human health. Symp Ser Soc Appl Microbiol 27: 35S–40S. 10.1046/j.1365-2672.1998.0840s135s.x 9750360

[32] Baggaley RF, Solomon AW, Kuper H, Polack S, Massae PA, et al (2006) Distance to water source and altitude in relation to active trachoma in Rombo district, Tanzania. Trop Med Int Health 11: 220–227. 16451347 10.1111/j.1365-3156.2005.01553.x PMC6855913

[33] Wang X, Hunter PR (2010) A systematic review and meta-analysis of the association between self-reported diarrheal disease and distance from home to water source. Am J Trop Med Hyg 83: 582–584. 20810824 10.4269/ajtmh.2010.10-0215 PMC2929055

[34] Kovats RS, Hajat S (2008) Heat stress and public health: a critical review. Annu Rev Public Health 29: 41–55. 18031221 10.1146/annurev.publhealth.29.020907.090843

[35] van Aalst MK (2006) The impacts of climate change on the risk of natural disasters. Disasters 30: 5–18. 16512858 10.1111/j.1467-9523.2006.00303.x

[36] Kovats RS (2003) Climate change, temperature and foodborne disease. Eurosurveillance 7: 2339.

[37] Lake IR, Gillespie IA, Bentham G, Nichols GL, Lane C, et al (2009) A re-evaluation of the impact of temperature and climate change on foodborne illness. Epidemiol Infect 137: 1538–1547. 19371450 10.1017/S0950268809002477

[38] World Health Organization Europe (2008) Protecting Health in Europe from climate change. Available: http://www.euro.who.int/__data/assets/pdf_file/0016/74401/E91865.pdf. Accessed 2013 Mar 24.

[39] Guyatt GH, Oxman AD, Schunemann HJ, Tugwell P, Knottnerus A (2011) GRADE guidelines: a new series of articles in the Journal of Clinical Epidemiology. J Clin Epidemiol 64: 380–382. 21185693 10.1016/j.jclinepi.2010.09.011

[40] Balshem H, Helfand M, Schunemann HJ, Oxman AD, Kunz R, et al (2011) GRADE guidelines: 3. Rating the quality of evidence. J Clin Epidemiol 64: 401–406. 21208779 10.1016/j.jclinepi.2010.07.015

[41] Erlanger TE, Keiser J, Utzinger J (2008) Effect of dengue vector control interventions on entomological parameters in developing countries: a systematic review and meta-analysis. Med Vet Entomol 22: 203–221. 18816269 10.1111/j.1365-2915.2008.00740.x

[42] Esu E, Lenhart A, Smith L, Horstick O (2010) Effectiveness of peridomestic space spraying with insecticide on dengue transmission; systematic review. Trop Med Int Health 15: 619–631. 20214764 10.1111/j.1365-3156.2010.02489.x

[43] Heintze C, Velasco Garrido M, Kroeger A (2007) What do community-based dengue control programmes achieve? A systematic review of published evaluations. Trans R Soc Trop Med Hyg 101: 317–325. 17084427 10.1016/j.trstmh.2006.08.007

[44] Ballenger-Browning KK, Elder JP (2009) Multi-modal Aedes aegypti mosquito reduction interventions and dengue fever prevention. Trop Med Int Health 14: 1542–1551. 19788717 10.1111/j.1365-3156.2009.02396.x

[45] Al-Muhandis N, Hunter PR (2011) The value of educational messages embedded in a community-based approach to combat dengue fever: A systematic review and meta regression analysis. Plos NTD 5: e1278. 10.1371/journal.pntd.0001278 PMC3160295 21886848

[46] Keiser J, Singer BH, Utzinger J (2005) Reducing the burden of malaria in different eco-epidemiological settings with environmental management: a systematic review. Lancet Infect Dis 5: 695–708. 16253887 10.1016/S1473-3099(05)70268-1

[47] Romero GA, Boelaert M (2010) Control of visceral leishmaniasis in latin america-a systematic review. PLoS Negl Trop Dis 4: e584. 20098726 10.1371/journal.pntd.0000584 PMC2808217

[48] Choi HW, Breman JG, Teutsch SM, Liu S, Hightower AW, et al (1995) The effectiveness of insecticide-impregnated bed nets in reducing cases of malaria infection: a meta-analysis of published results. Am J Trop Med Hyg 52: 377–382. 7771600 10.4269/ajtmh.1995.52.377

[49] Gamble C, Ekwaru PJ, Garner P, ter Kuile FO (2007) Insecticide-treated nets for the prevention of malaria in pregnancy: a systematic review of randomised controlled trials. PLoS Med 4: e107. 17388668 10.1371/journal.pmed.0040107 PMC1831739

[50] Eisele TP, Larsen D, Steketee RW (2010) Protective efficacy of interventions for preventing malaria mortality in children in Plasmodium falciparum endemic areas. Int J Epidemiol 39 Suppl 1i88–101. 20348132 10.1093/ije/dyq026 PMC2845865

[51] Lawrance CE, Croft AM (2004) Do mosquito coils prevent malaria? A systematic review of trials. J Travel Med 11: 92–96. 15109473 10.2310/7060.2004.17015

[52] Graves P, Gelband H (2006a) Vaccines for preventing malaria (SPf66). Cochrane Database of Systematic Reviews: CD005966. 10.1002/14651858.CD005966 PMC6532709 16625647

[53] Graves P, Gelband H (2006b) Vaccines for preventing malaria (blood-stage). Cochrane Database of Systematic Reviews: CD006199. 10.1002/14651858.CD006199 PMC6532641 17054281

[54] Graves P, Gelband H (2006c) Vaccines for preventing malaria (pre-erythrocytic). Cochrane database of systematic reviews (Online). 10.1002/14651858.CD006198 PMC6532586 17054280

[55] Demicheli V, Debalini MG, Rivetti A (2009) Vaccines for preventing tick-borne encephalitis. Cochrane Database of Systematic Reviews. 10.1002/14651858.CD000977.pub2 PMC6532705 19160184

[56] Warshafsky S, Lee DH, Nadelman RB, Wormser GP (2010) Efficacy of antibiotic prophylaxis for the prevention of Lyme disease: an updated systematic review and meta-analysis-author's response. Journal of Antimicrobial Chemotherapy 65: 2271–2273. 20382722 10.1093/jac/dkq097

[57] Garner P, Gulmezoglu AM (2003) Drugs for preventing malaria-related illness in pregnant women and death in the newborn. Cochrane Database Syst Rev: CD000169. 10.1002/14651858.CD000169 12535391

[58] Carmona-Fonseca J (2006) High efficacy of primary chemoprophylaxis with primaquine. Metanalysis. Iatreia 19: 244–260.

[59] Croft A, Garner P (1997) Mefloquine to prevent malaria: a systematic review of trials. British Medical Journal 315: 1412–1416. 9418088 10.1136/bmj.315.7120.1412 PMC2127902

[60] Nakato H, Vivancos R, Hunter PR (2007) A systematic review and meta-analysis of the effectiveness and safety of atovaquone proguanil (Malarone) for chemoprophylaxis against malaria. Journal of Antimicrobial Chemotherapy 60: 929–936. 17848375 10.1093/jac/dkm337

[61] ter Kuile FO, van Eijk AM, Filler SJ (2007) Effect of sulfadoxine-pyrimethamine resistance on the efficacy of intermittent preventive therapy for malaria control during pregnancy: a systematic review. JAMA 297: 2603–2616. 17579229 10.1001/jama.297.23.2603

[62] Garner P, Gulmezoglu AM (2006) Drugs for preventing malaria in pregnant women. Cochrane Database Syst Rev: CD000169. 10.1002/14651858.CD000169.pub2 17054128

[63] Meremikwu MM, Donegan S, Esu E (2008) Chemoprophylaxis and intermittent treatment for preventing malaria in children. Cochrane Database of Systematic Reviews: CD003756. 10.1002/14651858.CD003756.pub2 16235340

[64] Arnold BF, Colford JM Jr (2007) Treating water with chlorine at point-of-use to improve water quality and reduce child diarrhea in developing countries: a systematic review and meta-analysis. American Journal of Tropical Medicine & Hygiene 76: 354–364. 17297049

[65] Cairncross S, Hunt C, Boisson S, Bostoen K, Curtis V, et al.. (2010) Water, sanitation and hygiene for the prevention of diarrhoea. International Journal of Epidemiology 39 Suppl 1. 10.1093/ije/dyq035 PMC2845874 20348121

[66] Clasen T, Schmidt W-P, Rabie T, Roberts I, Cairncross S (2007) Interventions to improve water quality for preventing diarrhoea: systematic review and meta-analysis. BMJ 334: 782. 17353208 10.1136/bmj.39118.489931.BE PMC1851994

[67] Fewtrell L, Kaufmann RB, Kay D, Enanoria W, Haller L, et al (2005) Water, sanitation, and hygiene interventions to reduce diarrhoea in less developed countries: A systematic review and meta-analysis. Lancet Infectious Diseases 5: 42–52. 15620560 10.1016/S1473-3099(04)01253-8

[68] Gundry S, Wright J, Conroy R (2004) A systematic review of the health outcomes related to household water quality in developing countries. Journal of Water and Health 2: 1–13. 15384725

[69] Hunter PR (2009) Household Water Treatment in Developing Countries: Comparing Different Intervention Types Using Meta-Regression. Environmental Science & Technology 43: 8991–8997. 19943678 10.1021/es9028217

[70] Waddington H, Snilstveit B, White H, Fewtrell L (2009) Water, sanitation and hygiene interventions to combat childhood diarrhoea in developing countries. Available: http://www.dfid.gov.uk/R4D//PDF/Articles/SR_Sanitation.pdf. Accessed 2013 Mar 24.

[71] Graves PM, Deeks JJ, Demicheli V, Jefferson T (2010) Vaccines for preventing cholera: killed whole cell or other subunit vaccines (injected). Cochrane Database of Systematic Reviews 8: CD000974. 10.1002/14651858.CD000974.pub2 PMC6532721 20687062

[72] Bowler DE, Buyung-Ali L, Knight TM, Pullin AS (2010) Urban greening to cool towns and cities: A systematic review of the empirical evidence. Landscape and Urban Planning 97: 147–155.

[73] Bassil KL, Cole DC (2010) Effectiveness of public health interventions in reducing morbidity and mortality during heat episodes: a structured review. Int J Environ Res Public Health 7: 991–1001. 20617014 10.3390/ijerph7030991 PMC2872323

[74] Murphy BR, Whitehead SS (2011) Immune response to dengue virus and prospects for a vaccine. Annu Rev Immunol 29: 587–619. 21219187 10.1146/annurev-immunol-031210-101315

[75] Fletcher RW, Fletcher SW (2005) Clinical epidemiology the essentials 4th edn. Baltimore: Lippincott Williams and Wilkins. 140 p.

[76] Streiner DL, Norman GR (1998) PDQ Epidemiology 2nd edn. Ontario: BC Dekker Inc, Hamilton.

[77] Schmidt WP, Cairncross S (2009) Household water treatment in poor populations: is there enough evidence for scaling up now? Environ Sci Technol 43: 986–992. 19320147 10.1021/es802232w

[78] Hunter PR (2010) Apparent benefit of water filters may be an artifact of study design. Am J Public Health 100: 1557–1558; author reply 1558–1559. 10.2105/AJPH.2010.194621 PMC2920961 20671259

[79] Zmirou D, Rey S, Courtois J-P, Ferley J-P, Blatier J-F, et al (1995) Residual health risk after simple chlorine treatment of drinking water in small community systems. European Journal of Public Health 5: 75–81.

[80] Hunter PR, Zmirou-Navier D, Hartemann P (2009) Estimating the impact on health of poor reliability of drinking water interventions in developing countries. Science of the Total Environment 407: 2621–2624. 19193396 10.1016/j.scitotenv.2009.01.018

[81] Bouchama A, Dehbi M, Mohamed G, Matthies F, Shoukri M, et al (2007) Prognostic factors in heat wave-related deaths – A meta-analysis. Archives of Internal Medicine 167: 2170–2176. 17698676 10.1001/archinte.167.20.ira70009

[82] Abrahamson V, Wolf J, Lorenzoni I, Fenn B, Kovats S, et al (2009) Perceptions of heatwave risks to health: interview-based study of older people in London and Norwich, UK. J Public Health (Oxf) 31: 119–126. 19052099 10.1093/pubmed/fdn102

