# Supplementary material for: The Effectiveness of Public Health Interventions to Reduce the Health Impact of Climate Change: A Systematic Review of Systematic Reviews
Source: PLoS One. 2013 Apr 25;8(4):e62041. doi: 10.1371/journal.pone.0062041 (PMC3636259; doi:10.1371/journal.pone.0062041)
Supplement: Table S1 — Summary of search and Index terms for each public health disease/area, the number of identified papers and papers included in analysis. (DOCX) [file pone.0062041.s001.docx]

Table S1: Summary of search and Index terms for each public health disease/area, the number of identified papers and papers included in analysis

| **Disease/ health risk** | **Key words/ MeSH terms** | **Total number of papers identified** | **Included papers (for analysis)** |
| --- | --- | --- | --- |
| Crimean-Congo haemorrhagic fever | Crimean congo hemorrhagic fever | 3 | **0** |
| Chikungunya | Chikungunya | 4 | **0** |
| Cholera | Cholera, vibrio, vulnificus, parahaemolyticus | 107 | **1** |
| Cyanobacteria | Cyanobacteria, blue green algae | 18 | **0** |
| Dengue | Dengue, dengue hemorrhagic fever, dengue virus | 55 | **5** |
| Droughts | Drought | 39 | **0** |
| Floods | Flood, extreme weather | 79 | **0** |
| Heat stress | Extreme heat, hot temperature, heat exhaustion, body temperature regulation, heat stress disorders, thermogenesis, heat stroke, heat stress, heat wave, heat episode, heat event, extreme weather | 588 | **2** |
| Leishmaniasis | Leishmania | 74 | **1** |
| Lyme disease | Lyme disease, Lyme borreliosis | 57 | **1** |
| Malaria | Malaria, falciparum, vivax | 438 | **14** |
| RVF | Rift valley fever | 4 | **0** |
| Spotted fever rickettsioses | Spotted fever rickettsioses, boutonneuse fever, rocky mountain spotted fever, typhus | 8 | **0** |
| Tick borne encephalitis | Tick borne encephalitis | 13 | **1** |
| Water | Water | 1648 | **8** |
| West Nile | West Nile fever, West Nile virus | 23 | **0** |
| Yellow fever | Yellow fever | 18 | **0** |
| **Total** |  | 3176 | **33** |
