# Supplementary material for: The Effectiveness of Public Health Interventions to Reduce the Health Impact of Climate Change: A Systematic Review of Systematic Reviews
Source: PLoS One. 2013 Apr 25;8(4):e62041. doi: 10.1371/journal.pone.0062041 (PMC3636259; doi:10.1371/journal.pone.0062041)
Supplement: Table S2 — GRADE assessment of level of evidence for public health interventions to reduce the health impact of climate change. (DOC) [file pone.0062041.s002.doc]

Table S2: GRADE assessment of level of evidence for public health interventions to reduce the health impact of climate change

| **Intervention** | **Outcome** | **References** | **Risk of bias*** | **Inconsistency*** | **Indirectness of evidence*** | **Imprecision*** | **Publication bias**** | **Other issues***** | **GRADE summary score** |
| --- | --- | --- | --- | --- | --- | --- | --- | --- | --- |
| **Environmental interventions for control of vector-borne diseases** |  |  |  |  |  |  |  |  |  |
| **Dengue** |  |  |  |  |  |  |  |  |  |
| Outdoor insecticide spraying | Reduction in entomological parameters | Erlanger et al, 2008 | Intervention, 5 studies, Validity unclear, -2 | Results of the 5 studies clearly heterogeneous, -1 | Yes, no evidence on health outcomes, -1 | ~1700 houses, 0 | Undetected, 0 | None apparent, 0 | -4, very low quality, BEST: -1, moderate quality |
| Biological control of the vector (e.g. larvivorous fish, predatory insect larvae, copepods) | Reduction in entomological parameters | Erlanger et al, 2008 | Intervention, 9 studies, validity unclear, -2 | Results of the 9 studies clearly heterogeneous, -1 | Yes, no evidence on health outcomes, -1 | Unclear but >2000 houses, 0 | Strongly suspected, -1 | Large effect size, +1 | -4, very low quality, BEST: -1 moderate quality |
| Environmental management (e.g. removal of unused water vessels and covering of water containers) | Reduction in entomological parameters | Erlanger et al, 2008 | Intervention, 9 studies, validity unclear, -2 | Results of the 9 studies clearly heterogeneous, -1 | Yes, no evidence on health outcomes, -1 | Unclear but >2500 houses, 0 | Strongly suspected, -1 | None apparent, 0 | -5, very low quality, BEST: -2 low quality |
| Integrated vector management (environmental management combined with vector control) [Observational] | Reduction in entomological parameters | Erlanger et al, 2008 | Unclear, 18 studies, validity unclear, -2 | Results of the 18 studies clearly heterogeneous, -1 | Yes, no evidence on health outcomes, -1 | Sample size unclear but large, 0 | Unclear, -1 | Large effect size (all summary Res <0.5), +1 | -4, very low quality, BEST: 0 low quality |
| Peridomestic space spraying with insecticide | Dengue incidence (new dengue cases) | Esu et al, 2010 | Intervention, 1 before-after study, validity unclear, -2 (best -1) | Only one study assessed outcome, -1 | No, 0 | Number of events and participants unclear, -2 | Unclear, but <10 studies, 0 | None apparent, 0 | -5, very low quality, BEST: -2 low quality |
| Peridomestic space spraying with insecticide | Reduction in entomological indices | Esu et al, 2010 | Intervention, 1 RCT, 1 CCT, 13 before/after studies, validity unclear, -2 | 13/15 studies suggested protection, not further reported, -1 | Yes, no evidence on health outcomes, -1 | 187 houses, -1 | Unclear, -1 | None apparent, 0 | -6, very low quality, BEST: -3 very low quality |
| Community based dengue control programmes (education and/or insecticide spraying and/or biological control) | Entomological indices, detection of larval stages, confirmed dengue cases | Heintze et al, 2007 | Intervention, 2 RCTs, 6 before/after, 3 interrupted time series, validity unclear, -2 | Results of the 2 studies not consistent, -1 | Yes, only 1 study reported dengue incidence, -1 | >100,000 people, 0 | Unclear, -1 | None apparent, 0 | -5, very low quality, BEST: -1 moderate quality |
| Educational or behavioural interventions | Reduction in entomological parameters | Ballenger-Browning and Elder, 2009 | Intervention, 5 trials, validity unclear, -2 | Results of the studies not consistent though all suggested protection, -1 | Yes, no evidence on health outcomes, -1 | 187 houses + 13 4-block communities, 15 neighbourhoods, 0 | Unclear, but <10 studies, 0 | None apparent, 0 | -4, very low quality, BEST: -1 moderate quality |
| Biological intervention | Reduction in entomological parameters | Ballenger-Browning and Elder, 2009 | Intervention, 5 CTs, validity unclear, -2 | Results of the 5 studies consistent, 0 | Yes, no evidence on health outcomes, -1 | 66 containers & 12 communes & 553 houses, 0 | Unclear, but <10 studies, 0 | Large effect size (75-100% reduction), +1 | -2, low quality, BEST: 0 high quality |
| Insecticide spraying | Reduction in entomological parameters | Ballenger-Browning and Elder, 2009 | Intervention, 3 RCTs & 3 CTs, validity unclear, -2 | Results of the 6 studies not consistent, -1 | Yes, no evidence on health outcomes, -1 | 2112 houses, 0 | Unclear, but <10 studies, 0 | None apparent, 0 | -4, very low quality, BEST: -1 moderate quality |
| Community-based educational interventions | Reduction in entomological indices | Al-Muhandis and Hunter, 2011 | Intervention, 22 interventions with concurrent or historical controls, validity unclear, analysed effects of other methodological issues, -2 | Results not consistent and heterogeneity explored, 0 | Yes, no evidence on health outcomes, -1 | Sample size unclear but large, 0 | Unclear, -1 | None apparent, 0 | -4, very low quality, BEST: -1 moderate quality |
| **Malaria** |  |  |  |  |  |  |  |  |  |
| Environmental modification (to reduce vector habitats), long-lasting e.g. Installation/ maintenance of drains | Combined data on malaria incidence, spleen rates & mortality | Keiser et al, 2005 | Intervention, 27 of what appear to be before/after studies, validity unclear, -2 | Heterogeneity high, but not explored, though RRs consistently <1, -1 | No, 0 | Sample size unclear but large, -1 | Present (Eggers test -15, 95% CI -30 to -0.4), -1 | Large effect size (RR 0.12), +2 | -3, very low quality, BEST: 0 high quality |
| Environmental manipulation (temporary unfavourable vector conditions) e.g. water & vegetation management | Combined data on malaria incidence, spleen rates & mortality | Keiser et al, 2005 | Intervention, 4 before/after studies, validity unclear, -2 | Only 1 study had numerical data, -1 | No, 0 | Sample size ~500, cases unclear but >1000, 0 | Unclear, but <10 studies, 0 | Large effect size (RR 0.08), +2 | -1, moderate quality, BEST: +1 high quality |
| Modification of human habitation e.g. improving quality of houses | Combined data on malaria incidence, spleen rates & mortality | Keiser et al, 2005 | Intervention, 9 CCTs, validity unclear, -2 | Heterogeneity high, but not explored, though RRs consistently <1, -1 | No, 0 | No. of cases unclear but >700, 0 | Heterogeneity present, p<0.001, but not explored, -1 | Large effect size (RR 0.21), +1 | -3, very low quality, BEST: 0 high quality |
| **Leishmaniasis** |  |  |  |  |  |  |  |  |  |
| Dog culling | Human leishmaniasis or seroconversion | Romero and Boelaert, 2010 | Interventional, 3 intervention, 2 observational, Validity unclear, -2 | Heterogeneity not assessed, -1 | No, 0 | Human sero-conversion assessed in 2 intervention studies, but no. of cases unclear, -2 | <10 studies so not easy to assess, 0 | None apparent, 0 | -5, very low quality, BEST: 0 high quality |
| Use of insecticide | Human leishmaniasis | Romero and Boelaert | Interventional, 4 intervention, Validity unclear, -2 | Heterogeneity not assessed, -1 | No, 0 | No studies reported human leishmaniasis, -2 | <10 studies so not easy to assess, 0 | None apparent, 0 | -5, very low quality, BEST: -2 low quality |
| Combined interventions (dog culling and insecticide spraying) | Human leishmaniasis | Romero and Boelaert | Interventional, 2 RCT, 2 uncontrolled. Validity unclear, -2 | Heterogeneity not assessed, -1 | No, 0 | No. of participants and cases unclear, -2 | <10 studies so not easy to assess, 0 | None apparent, 0 | -5, very low quality, BEST: 0 high quality |
| Dog vaccine | Human leishmaniasis or seroconversion | Romero and Boelaert | Interventional, 3 intervention, 2 observational, Validity unclear, -2 | Heterogeneity not assessed, -1 | No, 0 | No human outcome data, -2 | <10 studies so not easy to assess, 0 | None apparent, 0 | -5, very low quality, BEST: -2 low quality |
| **Personal protective measures for control of vector-borne diseases (malaria)** |  |  |  |  |  |  |  |  |  |
| Insecticide-treated mosquito nets (ITN) | Clinical malaria (*Plasmodium falciparum* parasitemia) | Choi et al, 2003 | Intervention, 7 RCTs & 3 CCTs found, validity assessed but not reported, -2 | Heterogeneity apparent but not reported or discussed, -1 | No, 0 | Unclear, -2 | Not assessed, -1 | None apparent, 0 | -6, very low quality, BEST: 0 high quality |
| Insecticide-treated mosquito nets (ITN) in pregnancy | Mean haemoglobin levels (g/l) | Gamble et al, 2007 | Intervention, 5 RCTs, AC and incomplete accounting adequate for 3, blinding not mentioned, -1 | Little heterogeneity, 0 | No, 0 | No, >6000 participants, 0 | <10 studies so not easy to assess, 0 | None apparent, 0 | -1, moderate quality, BEST: 0 high quality |
| Insecticide-treated mosquito nets (ITN) in pregnancy | Mean birth weight (g) | Gamble et al, 2007 | Intervention, 5 RCTs, AC and incomplete accounting adequate for 3, blinding not mentioned, -1 | Little heterogeneity, 0 | No, 0 | No, >6000 participants, 0 | <10 studies so not easy to assess, 0 | None apparent, 0 | -1, moderate quality, BEST: 0 high quality |
| Insecticide-treated mosquito nets (ITN) in pregnancy | Miscarriages/ stillbirths | Gamble et al, 2007 | Intervention, 3 RCTs, AC and incomplete accounting adequate for 2, blinding not mentioned, -1 | No heterogeneity, 0 | No, 0 | Unclear but >4000 participants, -1 | <10 studies so not easy to assess, 0 | None apparent, 0 | -2, low quality, BEST: 0 high quality |
| Insecticide-treated mosquito nets (ITN) in pregnancy | Placental parasitemia | Gamble et al, 2007 | Intervention, 3 RCTs, AC & incomplete accounting adequate for 2, blinding not mentioned, -1 | Little heterogeneity, 0 | No, 0 | Unclear but >4000 participants, -1 | <10 studies so not easy to assess, 0 | None apparent, 0 | -2, low quality BEST: 0 high quality |
| Insecticide-treated mosquito nets (ITN) | All cause child mortality (ACCM)in children <5 years | Eisele et al, 2010 | Intervention, 3 community randomised RCTs, AC and follow up adequate, no blinding, -1 | No heterogeneity for ACCM | No, 0 | No, >3000 events, 0 | <10 studies so not easy to assess, 0 | None apparent, 0 | -1, moderate quality, BEST: -1 moderate quality |
| Insecticide-treated mosquito nets (ITN ) | Incidence of uncomplicated malaria in children <5 years | Eisele et al, 2010 | Intervention, 4 community randomised RCTs, AC and follow up adequate, no blinding, -1 | Some heterogeneity, not explored, -1 | No, 0 | No, >1500 events, 0 | <10 studies so not easy to assess, 0 | None apparent, 0 | -2, low quality, BEST: -1 moderate quality |
| Insecticide-treated mosquito nets (ITN ) | Prevalence of malaria parasitemia (in children <5 yrs) | Eisele et al, 2010 | Intervention, 6 community randomised RCTs, AC and follow up adequate, no blinding, -1 | Some heterogeneity, not explored, -1 | No, 0 | No, >4000 events, 0 | <10 studies so not easy to assess, 0 | None apparent, 0 | -2, low quality, BEST: -1 moderate quality |
| Indoor residual spraying (IRS) | All cause child mortality (ACCM,in children <5 yrs) | Eisele et al, 2010 | Intervention, 2 before-after studies, validity poor, -2 | Heterogeneity, not explored, -1 | No, 0 | No, >500 events, 0 | <10 studies so not easy to assess, 0 | None apparent, 0 | -3, very low quality, BEST: -2 low quality |
| Indoor residual spraying (IRS) | All cause infant mortality | Eisele et al, 2010 | Intervention, 3 before-after studies, validity poor, -2 | Heterogeneity, not explored, -1 | No, 0 | No, >700 events, 0 | <10 studies so not easy to assess, 0 | Large size effect (RR 0.47), +1 | -2, low quality, BEST: -1 moderate quality |
| Indoor residual spraying (IRS) | Incidence of uncomplicated malaria | Eisele et al, 2010 | Intervention, 3 before-after studies, validity poor, -2 | Heterogeneity, not explored, -1 | No, 0 | No, >1500 events, 0 | <10 studies so not easy to assess, 0 | Large size effect (RR 0.25), +1 | -2, low quality, BEST: -1 moderate quality |
| Indoor residual spraying (IRS) | Prevalence of malaria parasitemia (in children <5 yrs | Eisele et al, 2010 | Intervention, 5 before-after studies, validity poor, -2 | Heterogeneity, not explored, -1 | No, 0 | No, >3000 events, 0 | <10 studies so not easy to assess, 0 | Large size effect (RR 0.16), +2 | -1, moderate quality, BEST: 0 high quality |
| Intermittent preventive therapy (IPT) in pregnancy | Neonatal mortality | Eisele et al, 2010 | Intervention, 2 RCTs, AC adequate, 1 blinded, follow up not reported, -1 | No heterogeneity, 0 | No, 0 | Yes, 60 events, -2 | <10 studies so not easy to assess, 0 | None apparent, 0 | -3, very low quality, BEST: -2 low quality |
| Intermittent preventive therapy (IPT) in pregnancy | Perinatal mortality | Eisele et al, 2010 | Intervention, 1 RCT, validity not stated, -2 | Only 1 study assessed, -1 | No, 0 | Yes, 102 events, -1 | <10 studies so not easy to assess, 0 | None apparent, 0 | -4, very low quality, BEST: -2 low quality |
| Intermittent preventive therapy (IPT) and insecticide-treated mosquito nets (ITN) in pregnancy | Low birth weight | Eisele et al, 2010 | Intervention, 5 RCT, AC adequate, 2 blinded, follow up not reported, -2 (-1 best) | No heterogeneity, 0 | No, 0 | No, >300 events, 0 | <10 studies so not easy to assess, 0 | None apparent, 0 | -2, low quality, BEST: -1 moderate quality |
| Efficacy of mosquito coils to prevent malaria | Clinical malaria | Lawrance and Croft, 2006 | Intervention, No CCTs found | Heterogeneity not relevant | No, 0 | No events | None | None apparent | No evidence |
| Efficacy of mosquito coils to prevent malaria | Anti-mosquito outcome: bite reduction | Lawrance and Croft, 2006 | Intervention, 13 CCTs found, validity not reported, -2 | Not pooled (13 studies reported “positive” effect, 2 studies found no effect or did not report outcome (unclear)) – unable to assess, -1 | Yes, -1 | Unclear, -2 | Not assessed, -1 | None apparent | -7, very low quality, BEST: -1 moderate quality |
| Efficacy of mosquito coils to prevent malaria | Anti-mosquito outcome: repellence | Lawrance and Croft, 2006 | Intervention, 7 CCTs found, validity not reported, -2 | Not pooled (7 studies reported “positive” effect, 8 studies found no effect or did not report outcome (unclear)) – unable to assess, -1 | Yes, -1 | Unclear, -2 | Not assessed, -1 | None apparent | -7, very low quality, BEST: -1 moderate quality |
| Efficacy of mosquito coils to prevent malaria | Anti-mosquito outcome: percentage of mosquito mortality | Lawrance and Croft, 2006 | Intervention, 7 CCTs found, validity not reported, -2 | Not pooled (7 studies reported “positive” effect, 8 studies found no effect or did not report outcome (unclear)) – unable to assess, -1 | Yes, -1 | Unclear, -2 | Not assessed, -1 | None apparent | -7, very low quality, BEST: -1 moderate quality |
| **Immunization** **for vector-borne diseases** |  |  |  |  |  |  |  |  |  |
| **Malaria** |  |  |  |  |  |  |  |  |  |
| SPf66 Vaccine vs. placebo for preventing malaria | New malaria episode (*P. falciparum*) | Graves and Gelband, 2006a | Intervention, 9 RCTs, adequate AC in 8, all stated double blind, 0 | Heterogeneity, but explored, 0 | No, 0 | >2000 events, 0 | <10 studies so not easy to assess, 0 | None apparent, 0 | 0, high quality, BEST: 0 high quality |
| SPf66 Vaccine vs. placebo for preventing malaria | New malaria episode (*P. vivax*) | Graves and Gelband, 2006a | Intervention, 5 RCTs, adequate AC in 4, all stated double blind, 0 | Heterogeneity, but explored, 0 | No, 0 | >600 events, 0 | <10 studies so not easy to assess, 0 | None apparent, 0 | 0, high quality, BEST: 0 high quality |
| Blood stage Vaccine vs. placebo for preventing malaria | New malaria episode | Graves and Gelband, 2006b | Intervention, 1 RCT, adequate AC, blinding, follow up, 0 | Heterogeneity explored, 0 | No,0 | 49 events, -2 | <10 studies so not easy to assess, 0 | None apparent, 0 | -2 low quality, BEST: -2 low quality |
| CS-NANP Pre-erythrocytic vaccine vs. placebo for preventing malaria | New malaria episode | Graves and Gelband, 2006c | Intervention, 3 RCTs, 1 adequate AC, all double blind and good follow up, 0 | No heterogeneity, 0 | No, 0 | 112 events, -1 | <10 studies so not easy to assess, 0 | None apparent, 0 | -1, moderate quality, BEST: -1 moderate quality |
| RTS.S Pre-erythrocytic vaccine vs. placebo for preventing malaria | New malaria episode | Graves and Gelband, 2006c | Intervention, 3 RCTs, 2 adequate AC, all double blind and good follow up, 0 | Heterogeneity explored, 0 | No, 0 | 484 events, 0 | <10 studies so not easy to assess, 0 | None apparent, 0 | 0, high quality, BEST: 0 high quality |
| RTS.S Pre-erythrocytic vaccine vs. placebo for preventing malaria | Clinical malaria | Graves and Gelband, 2006c | Intervention, 2 RCTs, all adequate AC, double blind and good follow up, 0 | Heterogeneity explored, 0 | No, 0 | 531 events, 0 | <10 studies so not easy to assess, 0 | None apparent, 0 | 0, high quality, BEST: 0 high quality |
| ME-TRAP Pre-erythrocytic vaccine vs. placebo for preventing malaria | New malaria or clinical malaria | Graves and Gelband, 2006c | Intervention, 1 RCT, adequate AC, double blind and good follow up, 0 | No heterogeneity, 0 | No, 0 | ~112 events, -1 | <10 studies so not easy to assess, 0 | None apparent, 0 | -1, moderate quality, BEST: -1 moderate quality |
| **Tick-borne encephalitis** |  |  |  |  |  |  |  |  |  |
| Vaccines for preventing tick borne encephalitis (TBE) | TBE, antibody titre and adverse effects | Demichelli et al, 2009 | Interventional, 11 RCT & CCTs, AC unclear in all but 1 trial, single & double blinding plus open trials, drop-outs good, -1 | Heterogeneity (studies too varied to pool), -1 | No, 0 | No cases of TBE, -2 | Not assessed, -1 | None apparent, 0 | -5, very low quality, BEST: -3 very low quality |
| **Chemoprophylaxis** **for vector-borne diseases** |  |  |  |  |  |  |  |  |  |
| **Lyme disease** |  |  |  |  |  |  |  |  |  |
| Efficacy of antibiotic prophylaxis for the prevention of Lyme disease after *Ixodes* tick bite | Development of Lyme disease symptoms and presence of anti *Borrelia* antibodies | Warshafsky et al, 2010 | Interventional, 4 RCTs, AC not assessed but blinding & drop-outs good, -1 | No heterogeneity, 0 | No, 0 | 1145 randomised, 13 cases, -2 | <10 studies so not easy to assess, 0 | None apparent, 0 | -3, very low quality, BEST: -2 low quality |
| **Malaria** |  |  |  |  |  |  |  |  |  |
| Primary chemoprophylaxis with primaquine vs. placebo | Proportion of protection (no malaria) | Carmona-Fonseca, 2006 | Intervention, 7 CCTs, validity unclear, -2 | Some heterogeneity apparent in forest plot, not discussed or explored, -1 | No, 0 | >1000 participants, unclear how many malarial events, -1 | <10 studies so not easy to assess, 0 | None apparent, 0 | -4, very low quality, BEST: 0 high quality |
| Mefloquine | Malaria, and tolerability | Croft and Garner, 1997 | Intervention, 1 RCT for malaria, 10 for tolerability, AC, blinding & fate of participants good, 0 | Cannot assess heterogeneity for malaria, -1 | No, 0 | 53 cases from 1 trial, but clearly statistically significant effect (all cases in control group), -1 | <10 studies so not easy to assess, 0 | None apparent, 0 | -2, low quality, BEST: -1 moderate quality |
| Atovaquone- proguanil (Malarone) | Parasitaemia | Nakato et al., 2007 | Intervention, 5 RCTs, good validity, 0 | No heterogeneity, 0 | No, 0 | Unclear how many events, but large sample, -1 | <10 studies so not easy to assess, 0 | None apparent, 0 | -1, moderate quality, BEST: 0 high quality |
| Atovaquone- proguanil (Malarone) | Self- reported adverse effect | Nakato et al., 2007 | Intervention, 4 RCTs, good validity, 0 | Some heterogeneity, -1 | No, 0 | Unclear how many events, but large sample, -1 | <10 studies so not easy to assess, 0 | None apparent, 0 | -2, low quality, BEST: 0 high quality |
| intermittent sulfadoxine-pyrimethamine vs. placebo for malaria control during pregnancy | Placental malaria | ter Kuile et al., 2007 | Intervention, 4 RCTs, 1 with adequate AC, 1 with adequate follow up, 3 appeared blinded, -1 | Some heterogeneity but explored (subgrouping), 0 | No, 0 | >300 events, 0 | <10 studies so not easy to assess, 0 | RR<0.50, +1 | 0, high quality, BEST: 0 high quality |
| intermittent sulfadoxine-pyrimethamine vs. placebo for malaria control during pregnancy | Low birth weight (<2500g) | ter Kuile et al., 2007 | Intervention, 3 RCTs, 0 with adequate AC or adequate follow up, 2 appeared blinded, -2 | No heterogeneity, 0 | No, 0 | ~220 events, -1 | <10 studies so not easy to assess, 0 | RR<0.50, +1 | -2, low quality, BEST: -2 low quality |
| intermittent sulfadoxine-pyrimethamine vs. placebo for malaria control during pregnancy | Maternal anaemia | ter Kuile et al., 2007 | Intervention, 3 RCTs, 1 with adequate AC and follow up, 2 appeared blinded, -1 | Some heterogeneity but explored (subgrouping), 0 | No, 0 | >2000 events, 0 | <10 studies so not easy to assess, 0 | None apparent, 0 | -1, moderate quality, BEST: -1 moderate quality |
| intermittent sulfadoxine-pyrimethamine vs. monthly therapy for malaria control during pregnancy | Placental malaria | ter Kuile et al., 2007 | Intervention, 3 RCTs, 2 with adequate AC and moderate follow up, 1 appeared blinded, -1 | Some heterogeneity but explored (subgrouping), 0 | No, 0 | >300 events, 0 | <10 studies so not easy to assess, 0 | None apparent (only in subgroups), 0 | -1, moderate quality, BEST: -1 moderate quality |
| intermittent sulfadoxine-pyrimethamine vs. monthly therapy for malaria control during pregnancy | Mean birth weight (in Grams) | ter Kuile et al., 2007 | Intervention, 3 RCTs, 2 with adequate AC and moderate follow up, 1 appeared blinded, -1 | Some heterogeneity but explored (subgrouping), 0 | No, 0 | ~900 women, 0 | <10 studies so not easy to assess, 0 | None apparent, 0 | -1, moderate quality, BEST: -1 moderate quality |
| Drugs for preventing malaria vs. no drug in pregnant women | Antenatal parasitaemia | Garner and Gulmezoglu, 2006 | Intervention, 2 RCTs, both inadequate AC, no blinding, -2 | No heterogeneity, 0 | No, 0 | 57 events, -2 | <10 studies so not easy to assess, 0 | None apparent, 0 | -4, very low quality, BEST: -4 very low quality |
| Drugs for preventing malaria vs. no drug in pregnant women | Placental malaria | Garner and Gulmezoglu, 2006 | Intervention, 3 RCTs, all inadequate AC, no blinding, -2 | Clear heterogeneity, -1 | No, 0 | 195 events, -1 | <10 studies so not easy to assess, 0 | RR<0.5, +1 | -3, very low quality, BEST: -2 low quality |
| Drugs for preventing malaria vs. no drug in pregnant women | Antenatal anaemia | Garner and Gulmezoglu, 2006 | Intervention, 1 RCT, adequate AC and blinding, 0 | Heterogeneity not possible, -1 | No, 0 | 201 events, -1 | <10 studies so not easy to assess, 0 | None apparent, 0 | -2, low quality, BEST: -2 low quality |
| Drugs for preventing malaria vs. no drug in pregnant women | Perinatal death | Garner and Gulmezoglu, 2006 | Intervention, 4 RCTs, 1 with adequate AC and blinding, -1 | No heterogeneity, 0 | No, 0 | 130 events, -1 | <10 studies so not easy to assess, 0 | None apparent, 0 | -2, low quality, BEST: -2 low quality |
| Drugs for preventing malaria in pregnant women | Mean birth weight (g) | Garner and Gulmezoglu, 2006 | Intervention, 4 RCTs, 1 with adequate AC and blinding, -1 | Clear heterogeneity, -1 | No, 0 | >2600 participants, 0 | <10 studies so not easy to assess, 0 | None apparent, 0 | -2, low quality, BEST: -1 moderate quality |
| Drugs for preventing malaria in pregnant women | Low birth weight | Garner and Gulmezoglu, 2006 | Intervention, 2 RCTs, 1 with adequate AC and blinding, -1 | No heterogeneity, 0 | No, 0 | 229 events, -1 | <10 studies so not easy to assess, 0 | None apparent, 0 | -2, low quality, BEST: -2 low quality |
| Chemoprophylaxis and intermittent treatment with anti-malaria drugs for preventing malaria in children | Clinical malaria | Meremikwu et al., 2008 | Intervention, 10 RCTs, half with adequate AC, most were blinded, loss to follow-up often unclear, -1 | Clear heterogeneity, not explored, but all point estimates suggested protection, -1 | No, 0 | >2000 events, 0 | Authors stated that there were too few trials to assess publication bias in any forest plot, 0 | None apparent, 0 | -2, low quality, BEST: -1 moderate quality |
| Chemoprophylaxis and intermittent treatment with anti-malaria drugs | Severe anaemia | Meremikwu et al., 2008 | Intervention, 9 RCTs, half with adequate AC, most blinded, loss to follow-up often unclear, -1 | Some heterogeneity, not explored, but all point estimates suggested protection, -1 | No, 0 | >400 events, 0 | Authors stated that there were too few trials to assess publication bias in any forest plot, 0 | None apparent, 0 | -2, low quality, BEST: -1 moderate quality |
| Chemoprophylaxis and intermittent treatment with anti-malaria drugs | Hospital admission for any cause | Meremikwu et al., 2008 | Intervention, 10 RCTs, half with adequate AC, most were blinded, loss to follow-up often unclear, -1 | No heterogeneity, 0 | No, 0 | 108 events, -1 | Authors stated that there were too few trials to assess publication bias in any forest plot, 0 | None apparent, 0 | -2, low quality, BEST: -2 low quality |
| **Water interventions for control of waterborne diseases** |  |  |  |  |  |  |  |  |  |
| Relationship between diarrhoea and distance from home to water source [Observational] | Self reported diarrhoea | Wang and Hunter, 2010 | Observational, 6 studies, 4 prospective, 2 adjusted for confounders, -2 | Clear heterogeneity, explored, some explained, 0 | No, 0 | Unclear, but 5803 participants, -1 | <10 studies so not easy to assess, 0 | None apparent, 0 | -3, very low quality, BEST: -2 very low quality |
| Household chlorination of drinking water | Self reported diarrhoea | Arnold and Colford, 2007 | Intervention, 10 RCTs, validity unclear, -2 | Heterogeneity I^2^ 76%, explored, some explained, 0 | No, 0 | Unclear how many cases, 2802 households, -1 | Undetected, Begg test p=0.53, 0 | None apparent, 0 | -3, very low quality, BEST: 0 high quality |
| Water quality interventions | Self reported diarrhoea | Cairncross et al, 2010 | Intervention, 27 RCTs of which 4 blinded, other elements of validity not reported, -2 (-1) | Heterogeneity explored, 0 | No, 0 | Unclear, -2 | Not assessed, -1 | None apparent, 0 | -5, very low quality, BEST: -1 moderate quality |
| Impact of improvements in water quality (source and household interventions) | Self reported diarrhoea | Clasen et al, 2007 | Intervention, 22 RCTs (generally adequate AC and losses, but usually not blind) and 11 CCTs, -1 | Clear heterogeneity, explored, some explained, 0 | No, 0 | Unclear, cases not stated, >50,000 participants, -1 | Not assessed, -1 | None apparent, 0 | -3, very low quality, BEST: -1 moderate quality |
| Water supply interventions [Observational] | Self reported diarrhoea | Fewtrell et al, 2005 | Unclear, 9 studies, of which 6 described, but methodology not clear, -2 | Heterogeneity p<0.01, -1 | No, 0 | Unclear, no. of events not reported, -2 | Undetected (unclear how assessed), 0 | None apparent, 0 | -5, very low quality, BEST: 0 low quality |
| Water quality intervention at source [Observational] | Self reported diarrhoea | Fewtrell et al, 2005 | 3 studies, but methodology not stated, -2 | Unclear, -1 | No, 0 | Unclear, no. of events not reported, -2 | <10 studies so not easy to assess, 0 | None apparent, 0 | -5, very low quality, BEST: 0 low quality |
| Household water treatment | Self reported diarrhoea | Fewtrell et al, 2005 | 13 studies, but methodology not stated, -2 | Heterogeneity p<0.01, -1 | No, 0 | Unclear, no. of events not reported, -2 | Evidence detected (unclear how assessed), -1 | None apparent, 0 | -6, very low quality, BEST: -1 moderate quality |
| Household water interventions (water treatment and storage intervention) | Onset of diarrhoea | Gundry et al, 2004 | Intervention, 9 studies of which 4 blinded, no other elements of validity reported, -2 (-1) | Clear heterogeneity, explored, some explained, 0 | No, 0 | Unclear, -2 | No evidence, 0 | None apparent, 0 | -4, very low quality, BEST: -1 moderate quality |
| Household water interventions (water treatment & storage int.) | Onset of cholera | Gundry et al, 2004 | Intervention, 3 studies of which 0 blinded, no other elements of validity reported, -2 | No apparent heterogeneity, 0 | No, 0 | Unclear, -2 | No evidence, 0 | None apparent, 0 | -4, very low quality, BEST: 0 high quality |
| Household water treatment interventions | Self reported diarrhoea | Hunter, 2009 | Intervention, 28 RCTs, effects of blinding on outcome analysed, no other elements of validity reported, -2 | Heterogeneity explored, 0 | No, 0 | Unclear, -2 | Detected, Begg-Mazumdar test p=0.0012, -1 | None apparent, 0 | -5, very low quality, BEST: -1 moderate quality |
| Water supply intervention at point of use [Observational] | Self reported diarrhoea | Waddington et al, 2009 | Mixed, 0 RCTs, 5 studies, validity unclear, -2 | Clear heterogeneity, some explained, 0 | No, 0 | Unclear how many events, 52,000 participants, -1 | Publication bias strongly suggested, -1 | None apparent, 0 | -4, very low quality, BEST: -1 very low quality |
| Community water supply intervention at source | Self reported diarrhoea | Waddington et al, 2009 | Intervention, 0 RCTs, 2 studies, validity unclear, -2 | No heterogeneity, 0 | No, 0 | Events unclear, 1100 participants, -1 | Publication bias strongly suggested, -1 | None apparent, 0 | -4, very low quality, BEST: -1 moderate quality |
| Point of use water quality intervention | Self reported diarrhoea | Waddington et al, 2009 | Intervention, 25 RCTs of 28 studies, AC, blinding etc unclear, -2 | Clear heterogeneity, explored, some explained, 0 | No, 0 | Events unclear, 12,000 participants, -1 | Publication bias strongly suggested, -1 | None apparent, 0 | -4, very low quality, BEST: -1 moderate quality |
| Community water quality intervention at source | Self reported diarrhoea | Waddington et al, 2009 | Intervention, 2 RCTs of 3 studies, AC, blinding etc unclear, -2 | Clear heterogeneity, some explained, 0 | No, 0 | Events unclear, 2500 participants, -1 | Publication bias strongly suggested, -1 | None apparent, 0 | -4, very low quality, BEST: -1 moderate quality |
| **Immunization for waterborne diseases (cholera)** |  |  |  |  |  |  |  |  |  |
| Vaccines for preventing cholera | Death from cholera | Graves et al, 2007 | Intervention, 4 RCTs & 1 CCT, Good allocation concealment and blinding, 0 | No apparent heterogeneity, 0 | No, 0 | 837,000 participants, 48 deaths, -2 | <10 studies so not easy to assess, 0 | None apparent, 0 | -2, low quality, BEST: -2 low quality |
| Vaccines for preventing cholera | Cholera cases | Graves et al, 2007 | Intervention, 16 trials (RCTs & CCTs), Good allocation concealment and blinding, 0 | Some heterogeneity, well explored, 0 | No, 0 | 2800 events, 0 | Not assessed, -1 | None apparent, 0 | -1, moderate quality, BEST: 0 high quality |
| **Interventions for heat stress related disorders** |  |  |  |  |  |  |  |  |  |
| Effectiveness of greening to decrease air temperature in urban areas [Observational] | Day time air temperature in parks and green areas | Bowler et al, 2010 | Observational, 74 studies of which 4 were interventional, validity unclear, -2 | Clear heterogeneity, p<0.001, some exploration, 0 | Yes, no evidence on health outcomes, -1 | Unclear how many comparisons within each study, -2 | Not assessed, -1 | None apparent, 0 | -6, very low quality, BEST: -1 very low quality |
| Effectiveness of greening to decrease air temperature in urban areas [Observational] | Night time air temperature in parks and green areas | Bowler et al, 2010 | Observational, No. of studies unclear, validity unclear, -2 | Clear heterogeneity, p<0.001, some exploration, 0 | Yes, no evidence on health outcomes, -1 | Unclear how many comparisons within each study, -2 | Not assessed, -1 | None apparent, 0 | -6, very low quality, BEST: -1 very low quality |
| Heat health warning systems [Observational] | Public awareness of extreme heat episode, changes in practices and reduction of mortality and morbidity | Bassil and Cole, 2010 | Observational, 14 cross-sectional, economic or regression analyses, validity unclear, -2 | Unclear, -1 | No for mortality and morbidity, Yes for other outcomes, -1 | Unclear how many comparisons within each study, -2 | Not assessed, -1 | None apparent, 0 | -7 for heat episodes and practice, very low quality, BEST: -1 very low quality. -6 for mortality, very low quality, BEST: 0 low quality |

*Each assessed on the scale “no” (score 0), “serious” (score -1) or “very serious” (score -2). Where scores appear in red this was because the downgrading was due to lack of reporting in the systematic review (and may or may not indicate that the underlying research base was at risk of bias). The basis of assessment was:

- Risk of bias: Allocation concealment (AC), lack of blinding, incomplete accounting for fate of participants and selective outcome reporting – where none were assessed “validity unclear” was stated
- Inconsistency: Unexplained heterogeneity of results
- Indirectness of evidence: Presence of an indirect comparison or indirect evidence (studies did not directly address the question)
- Imprecision: For dichotomous outcomes imprecision was indicated by <300 total events, for continuous outcomes by total population size <400

**Publication bias was assessed as “undetected” (score 0) or “strongly suspected” (score -1) on the basis that it has been assessed and no evidence of bias has been found

***Other issues would upgrade the evidence, and include: large effect size (RR>2 or <0.5 score +1, RR>5 or <0.2 score +2), confounders working against bias (score +1), and/or presence of a dose response (score +1)

**GRADE summary score was the addition of the previous scores.**

For evidence based on **intervention** studies: Score ≥0 equated to “high quality”, -1 “moderate quality”, -2 “low quality” and ≤-3 “very low quality”.

For evidence based on **observational** studies ([Observational]): Score ≥+2 equated to “high quality”, +1 “moderate quality”, 0 “low quality” and ≤-1 “very low quality”.

The “BEST” GRADE score was the best possible score of the underlying evidence (assuming that characteristics not reported in reviews were all ideal), the GRADE score removing where the score was downgraded due to the review being unclear.
